# A Predominant Role of AtEDEM1 in Catalyzing a Rate-Limiting Demannosylation Step of an Arabidopsis Endoplasmic Reticulum-Associated Degradation Process

**DOI:** 10.3389/fpls.2022.952246

**Published:** 2022-07-07

**Authors:** Jianjun Zhang, Yang Xia, Dinghe Wang, Yamin Du, Yongwu Chen, Congcong Zhang, Juan Mao, Muyang Wang, Yi-Min She, Xinxiang Peng, Li Liu, Josef Voglmeir, Zuhua He, Linchuan Liu, Jianming Li

**Affiliations:** ^1^State Key Laboratory for Conservation and Utilization of Subtropical Agro-Bioresources, South China Agricultural University, Guangzhou, China; ^2^Guangdong Key Laboratory for Innovative Development and Utilization of Forest Plant Germplasm, College of Forestry and Landscape Architecture, South China Agricultural University, Guangzhou, China; ^3^Department of Molecular, Cellular, and Developmental Biology, University of Michigan, Ann Arbor, MI, United States; ^4^University of Chinese Academy of Sciences, Beijing, China; ^5^The Center of Excellence for Molecular Plant Sciences, Chinese Academy of Sciences, Shanghai, China; ^6^Glycomics and Glycan Bioengineering Research Center, College of Food Science and Technology, Nanjing Agricultural University, Nanjing, China

**Keywords:** endoplasmic reticulum-associated degradation, N-glycan, protein degradation, BRASSINOSTEROID-INSENSITIVE 1, α1,2-mannosidase, α1,6-mannose residue

## Abstract

Endoplasmic reticulum-associated degradation (ERAD) is a key cellular process for degrading misfolded proteins. It was well known that an asparagine (N)-linked glycan containing a free α1,6-mannose residue is a critical ERAD signal created by Homologous to α-mannosidase 1 (Htm1) in yeast and ER-Degradation Enhancing α-Mannosidase-like proteins (EDEMs) in mammals. An earlier study suggested that two Arabidopsis homologs of Htm1/EDEMs function redundantly in generating such a conserved N-glycan signal. Here we report that the Arabidopsis *irb1* (*reversal of bri1*) mutants accumulate brassinosteroid-insensitive 1–5 (bri1–5), an ER-retained mutant variant of the brassinosteroid receptor BRI1 and are defective in one of the Arabidopsis Htm1/EDEM homologs, AtEDEM1. We show that the wild-type AtEDEM1, but not its catalytically inactive mutant, rescues *irb1-1*. Importantly, an insertional mutation of the Arabidopsis Asparagine-Linked Glycosylation 3 (ALG3), which causes N-linked glycosylation with truncated glycans carrying a different free α1,6-mannose residue, completely nullifies the inhibitory effect of *irb1-1* on bri1-5 ERAD. Interestingly, an insertional mutation in AtEDEM2, the other Htm1/EDEM homolog, has no detectable effect on bri1-5 ERAD; however, it enhances the inhibitory effect of *irb1-1* on bri1-5 degradation. Moreover, *AtEDEM2* transgenes rescued the *irb1-1* mutation with lower efficacy than *AtEDEM1*. Simultaneous elimination of *AtEDEM1* and *AtEDEM2* completely blocks generation of α1,6-mannose-exposed N-glycans on bri1-5, while overexpression of either *AtEDEM1* or *AtEDEM2* stimulates bri1-5 ERAD and enhances the *bri1-5* dwarfism. We concluded that, despite its functional redundancy with AtEDEM2, AtEDEM1 plays a predominant role in promoting bri1-5 degradation.

## Introduction

Endoplasmic reticulum-associated degradation (ERAD) is an essential part of a highly conserved ER-localized protein quality control (ERQC) system for removing ER-retained nonnative or mis-assembled proteins, which involves retrotranslocation through the ER membrane, ubiquitination by a membrane-anchored ubiquitin ligase (E3), and eventual degradation *via* the cytosolic proteasome (Preston and Brodsky, 2017). A key event of this process is selection of terminally-misfolded proteins from repairable misfolded proteins and folding intermediates. However, little is known about how eukaryotic cells execute this selection step. Recent studies in yeast and mammalian cells have shown that an asparagine (Asn)-linked glycan (N-glycan) containing an exposed α1,6-mannose (Man) residue on misfolded glycoproteins serves as a crucial ERAD signal that marks a terminally misfolded glycoprotein for degradation. Such a signal is generated through trimming a specific terminal α1,2-Man residue from N-linked Man_8_GlcNAc_2_ (GlcNAc, N-acetylglucosamine) glycans by the Homologous to α-mannosidase 1 (Htm1) and mammalian ER-degradation enhancing α-mannosidase-like proteins (EDEMs; Quan et al., 2008; Clerc et al., 2009; Hosokawa et al., 2010). The exposed α1,6-Man residue and its surrounding misfolded region are recognized by the yeast OS-9 (Yos9)/mammalian Osteosarcoma amplified 9 (OS-9) protein and the yeast HMG-CoA reductase degradation protein 3 (Hrd3)/mammalian Suppressor/enhancer of lin-12-like protein1 (Sel1L) protein, respectively (Xu and Ng, 2015). It is believed that Yos9/OS-9 and Hrd3/Sel1L work together to bring a committed ERAD client to the membrane-anchored E3 ligase Hrd1 (HRD1 in mammals) for ubiquitination and subsequent retrotranslocation into the cytosol for proteasomal degradation (Smith et al., 2011).

Although similar processes were known to exist in plants (Ceriotti and Roberts, 2006; Liu et al., 2011), our knowledge about a plant ERAD system still remains limited (Strasser, 2018). Recent discoveries of several Arabidopsis ERAD clients made Arabidopsis an attractive genetic model system to study the plant ERAD process (Jin et al., 2007; Hong et al., 2009, 2012; Li et al., 2009; Nekrasov et al., 2009; Baer et al., 2016). Among them are bri1-5 and bri1-9, which are mutant variants of BRASSINOSTEROID-INSENSITIVE 1 (BRI1), a well-studied surface receptor for the plant steroid hormone brassinosteroids (BRs; Li and Chory, 1997; Kinoshita et al., 2005). A Cys^69^-Tyr mutation in bri1-5 and a Ser^662^-Phe mutation in bri1-9 are thought to cause minor structural defects that are recognized by a highly conserved ER quality control (ERQC) mechanism in Arabidopsis. This ERQC consists of EMS-mutagenized bri1 suppressor 1 (EBS1), the Arabidopsis homolog of the mammalian UDP-glucose:glycoprotein glucosyltransferase (UGGT) capable of differentiating misfolded glycoproteins from their native conformers, and EBS2 (also known as calreticulin 3 or CRT3), a plant-specific member of the CRT/calnexin (CNX) family capable of high-affinity binding to a monoglucosylated N-glycan (Jin et al., 2007, 2009; Hong et al., 2008). The EBS1-EBS2 system and other chaperone-mediated ERQC mechanisms retain the two mutant bri1 proteins in the ER, leading to their eventual degradation *via* ERAD and a severe BR-insensitive dwarf phenotype (Jin et al., 2007, 2009).

It was previously shown that the protein abundance of these Arabidopsis ERAD clients could be greatly increased by treatment with kifunensine (Kif; Hong et al., 2008, 2009; Nekrasov et al., 2009; Saijo et al., 2009), a widely used inhibitor of α1,2-mannosidases including Htm1/EDEMs (Elbein et al., 1990), suggesting involvement of Man-trimming steps in the Arabidopsis ERAD process (Liu and Li, 2014). Further genetic and metabolic studies not only confirmed this phamacological finding but also concluded that the N-glycan signal for tagging an Arabidopsis ERAD client is conserved to be a free α1,6-Man residue-containing N-glycan (Hong et al., 2012). The Arabidopsis has two homologs of Htm1/EDEMs, AtEDEM1, and AtEDEM2 (known previously as MNS5 and MNS4 for α-mannosidase 5 and 4, respectively), and a previous reverse genetic investigation suggested that these two Htm1/EDEM homologs function redundantly in ERAD of bri1-5 as single mutation of either protein fails to suppress the *bri1-5* phenotype (Huttner et al., 2014b). However, it remains unknown whether AtEDEM1 and AtEDEM2 are required to generate the conserved N-glycan code on a known ERAD client. Here, we report a forward genetic study showing that despite functional redundancy of AtEDEM1/MNS5 and AtEDEM2/MNS4, loss-of-function mutations in AtEDEM1 alone could partially suppress the dwarf phenotype of *bri1-5* by weakly inhibiting bri1-5 degradation. We have found that AtEDEM2 could rescue the *irb1-1* mutation but with a lower efficacy than AtEDEM1, likely due to its weaker promoter and a slightly weaker biochemical activity. More importantly, the mass spectrometry-based N-glycan analyses coupled with linkage-specific mannosidases demonstarted the functional redundancy of AtEDEM1 and AtEDEM2 in removing the C-branch terminal α1,2-Man residue, thus exposing the ERAD-signaling α1,6-Man residue. Furthermore, our transgenic experiments indicated that the AtEDEM1/AtEDEM2-catalyzed creation of the N-glycan ERAD signal constitutes a major rate-limiting step of the bri1-5 ERAD pathway.

## Materials and Methods

### Plant Materials and Growth Conditions

All *Arabidopsis* mutants and transgenic lines used in this study are in Wassilewskija-2 (Ws-2) or Columbia-0 (Col-0) ecotype. All 6 *irb1* mutants were isolated from two large-scale EMS-mutagenesis-based genetic screens for extragenic suppressors of the Arabidopsis *bri1-5* mutant (in Ws-2 ecotype; Noguchi et al., 1999). The T-DNA insertional mutant *edem2-t* (*SALK_095857*, Col-0) was obtained from the Arabidopsis Biological Resource Center (ABRC) at Ohio State University and crossed with *bri1-*5 and *irb1-1 bri1-5*, while the T-DNA insertional mutant *alg3-t2* (*SALK_046061*; Col-0) was previously described (Hong et al., 2012). Methods for seed sterilization and conditions for plant growth were described previously (Li et al., 2001), and the hypocotyl elongation assays on BL-containing medium were carried out according to a previously described protocol (Neff et al., 1999).

### Map-Based Cloning of the *IRB1* Gene

The *irb1 bri1-5* mutant (ecotype Ws-2) was crossed with a *bri1-9* mutant (ecotype Col-0; Jin et al., 2007), and the resulting F1 plants were allowed for self-fertilization to generate several F2 mapping populations. Genomic DNAs from segregating F2 seedlings exhibiting the *irb1 bri1-5*-like morphology were extracted as previously described (Li and Chory, 1998) and used for PCR-based mapping using previously published simple sequence length polymorphism markers (Pacurar et al., 2012), and oligonucleotides listed in Supplementary Table S1.

### Construction of Plasmids and Generation of Transgenic Plants

A 5,279-bp genomic fragment of *At1g27520* containing 1,348-bp promoter and 591-bp 3′-untranscribed/untranslated region was PCR-amplified from the BAC T17H3 DNA obtained from ABRC using the *gAtEDEM1* primer set (Supplementary Table S1) and was cloned into BamHI/SalI-digested *pPZP212* vector (Hajdukiewicz et al., 1994). The resulting *gAtEDEM1* plasmid was subsequently used to perform a site-directed mutagenesis using the *AtEDEM1Mut* primer set (Supplementary Table S1) and the QuikChange II XL Site-Directed Mutagenesis kit (Agilent) to generate a *mgAtEDEM1* transgene that produced a glutamate(E)^134^-glutamine(Q) mutated catalytically-inactive variant of AtEDEM1 by the manufacturer’s recommended protocol. A 6,323-bp genomic fragment of *At5g43710* containing 1,586-bp promoter/5′-untranslated region and a 466-bp 3′-untranslated/untranscribed region was amplified from the BAC MQD19 DNA (also obtained from ABRC) using the *gAtEDEM2* primer set (Supplementary Table S1) and subsequently cloned into the XmaI/KpnI-digested *pPZP212* vector (Hajdukiewicz et al., 1994). A 1,811-bp coding sequence (CDS) fragment and a 1,872-bp CDS fragment containing the entire coding region of *AtEDEM1* and *AtEDEM2* were amplified from an *At1g27520* cDNA clone R19200 and an *At5g43710* cDNA clone G09215 (both were obtained from ABRC) using the primer sets, *cAtEDEM1GFP* and *cAtEDEM2GFP* (Supplementary Table S1), double digested with SpeI/XbaI and BamHI (depending on the introduced restriction sites on the primers), and subsequently cloned into the XbaI/BamHI-digested *pBRI1::BRI1-GFP* (Friedrichsen et al., 2000) to generate a *pBRI1::cAtEDEM1-GFP* plasmid and *a pBRI1::cAtEDEM2-GFP* plasmid, respectively. To generate non-tagged *pBRI1::cAtEDEM1/2* plasmids, the first cDNAs of the wild-type Arabidopsis seedlings and the *pBRI1cAtEDEM1* and *pBRI1cAtEDEM2* primer sets (see Supplementary Table S1) were used to amplify the CDS fragments of AtEDEM1/2, which were digested with BamHI/KpnI and subsequently cloned into the BamHI/KpnI-digested *pC1300pBRI1* plasmid, a modified *pCambia1300* vector (Leclercq et al., 2015) that contains a 1.6-kb BRI1 promoter fragment. To create transgenes of *pBRI1::At1g30000-GFP*, *pBRI1::At1g51590-GFP*, and *pBRI1::Htm1-GFP*, the first strand cDNAs of the wild-type Arabidopsis plants and yeast genomic DNAs were used to amplify the open-reading frames of *At1g30000* (*MNS3*), *At1g51590* (*MNS1*), and the yeast Htm1 with the *1g30000GFP*, *1g51590GFP*, and *Htm1GFP* primer sets (Supplementary Table S1), respectively. The PCR-amplified CDS fragments were digested with XbaI/SpeI and BamHI/BglII (depending on the introduced restriction sites of the primers) and subsequently cloned into the XbaI/BamHI-digested *pBRI1::BRI1-GFP* plasmid to generate *pBRI1::c1g30000-GFP*, *pBRI1::c1g51590-GFP*, and *pBRI1::Htm1-GFP* plasmids. A two-step cloning strategy was used to create the *pBRI1::bri1-5ED-GFP-HDEL* plasmid. The coding sequence of the BRI1’s extracellular domain was amplified from a *pBRI1::bri1-5-GFP* plasmid (Hong et al., 2008) using the *BRI1ED* primer set (Supplementary Table S1), digested with SpeI and BamHI, and cloned into the XbaI/BamHI-cut *pBRI1::BRI1-GFP* plasmid (Hong et al., 2008) to generate the *pBRI1::bri1-5ED-GFP* plasmid. The resulting plasmid was used as the DNA template to amplify the coding sequence of GFP with the *GFPHdel* primer set [Supplementary Table S1, its reverse primer containing coding sequence of the HDEL (histidine-aspartate-glutamate-leucine) ER-retrieval motif], which was subsequently digested with BamHI and KpnI and cloned into the BamHI/KpnI-digested *pBRI1::bri1-5-GFP* plasmid to create the *pBRI1::BRI1ED-GFP-HDEL* plasmid. The created transgenes were fully sequenced to ensure no PCR-introduced error and were individually transformed into various Arabidopsis lines or used for transient expression in tobacco leaves.

### Transient Expression and Confocal Microscopic Analysis of GFP-Tagged EDEM Fusion Proteins in Tobacco Leaves

The *pBRI1::cAtEDEM1-GFP*, *pBRI1::cAtEDEM2-GFP*, *pBRI1::At1g3000-GFP*, *pBRI1::At1g51590-GFP*, and *pSITE03-ER-RFP* (encoding a red fluorescent protein (RFP) tagged at its C-terminus with the ER-retrieval HDEL motif; Chakrabarty et al., 2007), and *p35S:p19* (encoding the p19 protein of tomato bushy stunt virus that was known for suppressing gene silencing; Voinnet et al., 2003) plasmids were cotransformed into leaves of 3-week-old tobacco (*Nicotiana benthamiana*) plants *via* an *Agrobacterium*-mediated infiltration method (Voinnet et al., 2003). Forty-eight hours after infiltration, the localization patterns of AtEDEM1-GFP or AtEDEM2-GFP and the ER-localized RFP-HDEL in the co-infiltrated tobacco leaf epidermal cells were examined using a Leica confocal laser-scanning microscope (TCS SP5 DM6000B) with an HCX PL APOCS 63X 1.30 glycerin lens and LAS AF software (Leica Microsystems). The GFP or RFP signal was excited by using the 488- or 543-nm laser light, respectively.

### Yeast Complementation Assay

The yeast strain ∆*htm1* carrying the *pDN436* plasmid (Ng et al., 2000) that encodes a HA-tagged CPY* (the ER-retained misfolded variant of the vacuolar carboxypeptidase Y) was provided by Amy Chang (University of Michigan). The coding sequence of the yeast Htm1 and AtEDEM1 were individually amplified from the yeast genomic DNA and the first-strand Arabidopsis cDNAs, respectively, and the resulting PCR fragments were used to replace the yeast *ALG9* fragment from the *pYEp352-ScALG9* expression plasmid (Frank and Aebi, 2005) to create *pYEp352-Htm1* and *pYEp352-AtEDEM1* plasmids following a previously described cloning strategy (Hong et al., 2009). After sequencing to ensure no PCR-introduced error, these two plasmids were individually transformed into the ∆*htm1* mutant yeast cells by a previously-published transformation protocol (Gietz and Woods, 2002). Yeast cells of the ∆*htm1* mutant strain and *pYEp352-Htm1*/*AtEDEM1*-transformed ∆*htm1* strains were grown to mid-log phase (OD_600_ = ~1.5) and treated with 100 μg/ml CHX (cycloheximide). Similar amounts of yeast cells were removed at 0, 1, 2, and 4 h after the CHX addition, collected by centrifugation on a bench-top microcentrifuge at room temperature, and resuspended in 1X yeast extraction buffer (0.3 M sorbitol, 0.1 M NaCl, 5 mM MgCl_2_, and 10 mM Tris, pH 7.4). After cell lysis by vigorous vortexing with glass beads, the resuspended yeast cells were mixed with 2X SDS sample buffer [100 mM Tris–HCl, pH 6.8, 4% (w/v) SDS; 0.2% (w/v) bromophenol blue, 20% (v/v) glycerol, and 200 mM β-mercaptoethanol], boiled for 10 min, and centrifuged for 10 min to remove insoluble cellular debris. The resulting supernatants were separated on 10% SDS/PAGE and analyzed by immunoblotting with an anti-HA antibody (10A5; Invitrogen).

### Protein Extraction and Immunoblot Analyses

Two or 4-week-old *Arabidopsis* seedlings treated with or without CHX (Sigma-Aldrich), Kif (Toronto Research Chemicals), or BL (brassinolide) (Chemiclones, Inc. Canada), or 3 g of agro-infiltrated tobacco leaves, were ground into fine powder in liquid nitrogen, resuspended in 2X SDS sample buffer, and boiled for 10 min. After 10 min centrifugation in a bench-top Eppendorf microcentrifuge at the top speed at room temperature to remove insoluble cellular debris, the clear supernatants were used immediately for immunoblot analysis or incubated with or without 1,000 U Endo Hf in 1X G5 buffer (New England Biolabs) for 1 h at 37°C. These treated protein samples were subsequently separated by 7% or 10% SDS-PAGE and analyzed by Coomassie Blue staining or by immunoblot with antibody raised against BRI1, GFP (632381, Clontech), ACTIN (CW0264, Beijing CWBio), and BRI1-EMS-SUPPRESSOR1 (BES1) (Mora-Garcia et al., 2004). Chemiluminescence immunoblot signals were visualized by X-ray films or by the Odyssey® Dlx Infrared Imaging System (LI-COR).

### RNA Isolation and Reverse Transcription-PCR

Total RNAs were isolated from 2-week-old *Arabidopsis* seedlings grown on ½ MS medium containing 1% sucrose and 0.8% phytagel (Sigma) as described previously (Li et al., 2001). For each RT-PCR experiment, 2 μg of total RNAs were reverse transcribed using the Invitrogen’s SuperScript First-Strand Synthesis System for RT-PCR according to the manufacturer’s recommended protocol. To analyze the transcripts of *IRB1/AtEDEM1* in *bri1-5* and *irb1-1 bri1-5* mutant backgrounds or the *AtEDEM2* transcription in wild-type Col-0 and the *edem2-t* insertional mutants, 0.5 μl of the first-strand cDNA reaction products was used as a template for PCR amplification with the primer sets shown in Supplementary Table S1. The *ACTIN2* transcript was amplified using the *ACTIN2* primer set (Supplementary Table S1) as a control. Amplified RT-PCR products were separated by 1% agarose gel, visualized by ethidium bromide staining, and photographed with a Gel Doc™ XR+ Gel Documentation system (Bio-Rad).

### Glycan Structure Analysis

Ten grams of 4-week-old soil-grown plants were collected, immediately ground in liquid nitrogen, and then dissolved in the protein extraction buffer [50 mM Tris–HCl, pH 7.5, 150 mM NaCl, 5 mM EDTA, 0.2% (v/v) Triton X-100 (Sigma), 0.2% (v/v) Nonidet P-40 (Roche), 1 mM phenylmethylsulfonyl fluoride (PMSF, Sigma-Aldrich), and a cOmplete™ protease inhibitor cocktail (Roche)]. After 15 min centrifugation at 10,000 × *g* to remove insoluble cellular debris, the supernatants were used to immunoprecipitate the GFP-tagged bri1-5ED using anti-GFP monoclonal antibody-conjugated-agarose (D153-8, MBL International Corporation). The immunoprecipitated proteins were further separated by SDS-PAGE. The bri1-5ED-GFP-HDEL protein bands in the gel slices were digested by 50 ng of trypsin (Promega) followed by chymotrypsin (Sigma) in 25 mM NH_4_HCO_3_, and the extracted peptides were subsequently analyzed by the data dependent LC–MS/MS on an Orbitrap Fusion Tribrid mass spectrometer (Thermo) coupled with ultraperformance nanoflow LC system (Waters) to identify the glycosylated BRI1 peptides following a previously published procedure (Ma et al., 2016). The identification of N-glycopeptides was achieved through parallel LC–MS/MS analyses of intact glycopeptides by the low-energy collision-induced dissociation (CID) and high-energy collision-induced dissociation (HCD) to determine both peptide sequences and their-associated glycan structures. To accurately validate the structures of N-glycans on bri1-5ED-GFP-HDEL, the immunoprecipitated bri1-5ED-GFP-HDEL was directly eluted from the beads in the glycine buffer (0.1 M glycine HCl pH 3.0), and neutralized in the Tris–HCl buffer (pH 7.5). The purified protein samples were subsequently dried, dissolved with 23 μl 500 mM NaH_2_PO_4_, 12.5 μl denaturing buffer [containing 1 M β-mercaptoethanol and 2% (w/v) SDS]. Following the glycosidase digestion with PNGase F (Prozyme), samples were fluorescence labeled with 2AB and then separated by hydrophilic interaction liquid chromatography (HILIC). N-glycans of each single chromato-graphic fraction were collected, dried, subjected to further digestion by highly-specific α1,3-(Qlyco, Nanjing, China), α1,6-(Qlyco, Nanjing, China), and α1,2/3/6-exomanno-sidases (Prozyme), and separated by ultra-performance liquid chromatography (UPLC) (Liu et al., 2016a).

### Sequence and Phylogeny Analysis

Forty-one unique protein sequences were downloaded from NCBI and aligned using a MUSCLE program (Edgar, 2004) at http://www.phylogeny.fr (Dereeper et al., 2008). These sequences include IRB1/AtEDEM1/MNS5 (NP_564288); AtEDEM2/MNS4 (NP_199184); XP_006307064.1 and XP_006282375.1 of *Capsella rubella*; XP_010322255.1 and XP_004236144.1 (*Solanum lycopersicum*); XP_003536208.1 and XP_003549640.1 (*Glycine max*); XP_002311656.2 and XP_024437663.1 (*Populus trichocarpa*), XP_008646239.1 and XP_008654408.1 (*Zea mays*), XP_015622855.1 and XP_015619333.1 (*Oryza sativa*); XP_003573110.1 and XP_003569932.1 (*Brachypodium distachyon*); KMZ61171 and KMZ61126.1 (*Zostera marina*); XP_020518838.1 and XP_006838875.1 (*Amborella trichopoda*); XP_024536391.1 and XP_002969801.2 (*Selaginella moellendorffii*); XP_024401298.1 (*Physcomitrella patens*); PTQ27873.1 and PTQ31295.1 (*Marchantia polymorpha*); GBG76786.1 (*Chara braunii*); GAQ84904.1 and GAQ88520.1 (*Klebsormidium nitens*); XP_005643868.1 and XP_005647098.1 (*Coccomyxa subellipsoidea C-169*); XP_001420019.1 (*Ostreococcus lucimarinus* CCE9901), XP_003081735.3 (*Ostreococcus tauri*), XP_003059643.1 (*Micromonas pusilla* CCMP1545), XP_002504119.1 (*Micromonas commode*), XP_005845108.1 (a partial polypeptide from *Chlorella variabilis*); PRW45694.1 (*Chlorella sorokiniana*); and XP_007514253.1 (*Bathycoccus Prasinos*). The two spruce EDEM sequences were obtained as translational products of sequenced mRNAs (GCHX01235049 and GCHX01346827) from *Picea glauca*. Yeast Htm1 (NP_012074) and the Arabidopsis At1g51590/MNS1 (OAP12316.1, one of the two Golgi-localized α1,2-mannosidase; Liebminger et al., 2009), were used as the outgroups to root the phylogeny tree. The aligned sequences were used to construct a phylogeny tree by the PhyML program (Guindon et al., 2010) with the bootstrapping (number of bootstraps: 100) procedure at http://www.phylogeny.fr, and the derived consensus tree was visualized with the TreeDyn program.^1^ The aligned amino acid sequences were used to obtain the conserved 430-amino-acid-long core domains of glycosylhydrolase family 47 (glyco_hydro_47), which were subsequently used to perform pairwise comparison to obtain their sequence identity and similarity. The glyco_hydro_47 domains of AtEDEM1/2, their homologs of rice, *Selaginella*, *Amborella*, and the liverwort, plus those of the three human EDEMs (EDEM1, NP_055489; EDEM2, NP_001341937; and EDEM3, NP_001306889) were aligned by the MUSCLE program at www.phylogeny.fr and the resulting aligned sequences were visualized by the BoxShade program at http://embnet.vital-it.ch/software/BOX_form.html.

## Results

### Isolation and Characterization of *irb1* Mutants

**Figure 1:**
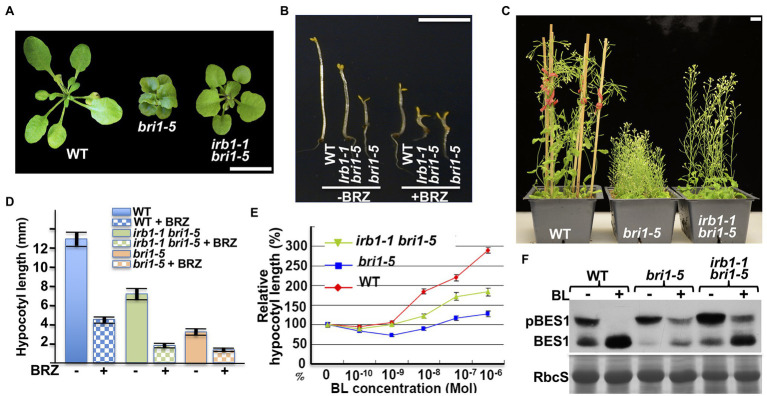
*irb1-1* suppresses the *bri1-5* phenotypes and confers partial BR sensitivity to the *bri1-5* mutant. **(A)** Photographs of 4-week-old soil-grown plants of the wild-type (ecotype Ws-2), *bri1-5*, and the *irb1-1 bri1-5* double mutant. **(B)** Photographs of 5-day-old dark-grown seedlings of wild-type (Ws-2), *irb1-1 bri1-5*, and *bri1-5* grown on medium supplement with or without 2 μM brassinazole (BRZ). **(C)** Photographs of 2-month-old soil-grown mature plants of wild-type, *bri1-5*, and *irb1-1 bri1-5*. In **(A–C)**, scale bar = 1 cm. **(D)** Quantitative analysis of average hypocotyl lengths of 5-day-old dark-grown seedlings grown on ½ MS medium supplemented with or without 2 μM BRZ. A total of ~60 seedlings from two independent experiments were analyzed and the error bars indicate ±SEs. **(E)** Quantitative analysis of hypocotyl elongation of 10-day-old light grown seedlings on ½ MS medium containing varying concentrations of brassinolide (BL). A total of ~90 seedlings from three biological replicates were analyzed. Each data point represents the relative value of average hypocotyl length of BL-treated seedlings to that of mock-treated seedlings of the same genotype, and error bars represent ±SEs. **(F)** Immunoblot analysis of BL-induced dephosphorylation of BES1. Equal amounts of total proteins extracted from 2-week-old light-grown seedlings treated with or without 1 μM BL for 2 h were separated by 10% SDS–PAGE and analyzed by immunoblotting using an anti-BES1 antibody. The lower strip is Coomassie blue staining of the small subunit of ribulose-1.5-bisphosphate carboxylase/oxygenase (RbcS) on a duplicated gel, which was used as a loading control.

The mutant bri1-5 receptor, which carries the Cys^69^-Tyr mutation in the extracellular domain of BRI1, is retained in the ER by at least three independent mechanisms and is degraded by a Kif-sensitive ERAD process (Hong et al., 2008). To identify components of its degradation machinery, we performed two large-scale ethyl methanesulfonate (EMS)-mutagenesis projects with the *bri1-5* mutant and isolated >60 *irb* mutants (*reversal of bri1-5*), several of which were found to be allelic to *ebs4*, *ebs5, ebs6,* and *ebs7* (Hong et al., 2009; Su et al., 2011, 2012; Liu et al., 2015). These screens also identified six allelic *irb1* mutants. As shown in Figures 1A–C, the *irb1-1* mutation nicely suppresses the growth defects of *bri1-5*. The *irb1-1 bri1-5* double mutant, compared to the parental *bri1-5* mutant, has a larger rosette with easily recognizable petioles (Figure 1A), a longer hypocotyl when grown in the dark (Figure 1B), and taller inflorescence stems at maturity (Figure 1C). Interestingly, the difference in etiolated hypocotyl length between *bri1-5* and *irb1-1 bri1-5* disappeared when grown on medium containing brassinazole (BRZ; Figures 1B,D), a specific inhibitor of BR biosynthesis (Asami et al., 2000), suggesting that the phenotypic suppression of *bri1-5* by *irb1-1* likely depends on BR perception. Consistent with these phenotypic changes, a BR-induced hypocotyl elongation assay (Neff et al., 1999) showed that *irb1-1* partially restored the BR sensitivity of the BR-insensitive mutant *bri1-5* (Figure 1E). An immunoblot assay that examined the BR-induced change in the phosphorylation status of BES1, a robust biochemical marker of BR signaling (Mora-Garcia et al., 2004), further supported increased BR sensitivity of *bri1-5* by *irb1-1* (Figure 1F).

### The *irb1-1* Mutation Inhibits the Degradation of bri1-5

**Figure 2:**
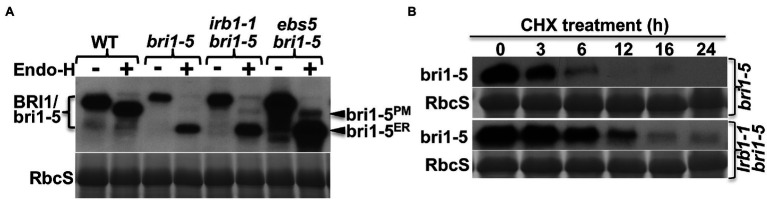
*irb1-1* is a weak endoplasmic reticulum-associated degradation (ERAD) mutant with increased abundance of bri1-5 **(A)**. Immunoblot analysis of the bri1-5 abundance. Equal amounts of total proteins extracted from 2-week-old light-grown seedlings were treated with or without Endo H, separated by 8% SDS–PAGE, and analyzed by immunoblotting using an anti-BRI1 antibody. **(B)** Immunoblot analysis of the bri1-5 stability in *bri1-5* and *irb1-1 bri1-5* mutants. Two-week-old seedlings were transferred into liquid ½ MS medium containing 180 μM cycloheximide (CHX). Equal amounts of seedlings were removed at indicated incubation times to extract total proteins in 2 X SDS sample buffer, which were subsequently analyzed by immunoblotting with the anti-BRI1 antibody. In both **(A,B)**, Coomassie blue staining of the RbcS band on duplicated gels was used as loading control.

Our previous studies showed that the restored BR sensitivity in suppressor mutants of two ER-retained BR receptors (bri1-5 and bri1-9) are caused by defective ER quality control (ERQC) systems including ERAD (Jin et al., 2007, 2009; Hong et al., 2008, 2009, 2012; Su et al., 2011, 2012; Liu et al., 2015). To determine if the *irb1-1* mutation inhibits ER retention or ERAD of bri1-5, we performed an immunoblot assay using an anti-BRI1 antibody (Mora-Garcia et al., 2004) and discovered that the *irb1-1 bri1-5* mutant accumulated more bri1-5 proteins than the parental *bri1-5* mutant (Figure 2A). However, the degree of the bri1-5 abundance increase was somewhat lower than what was observed in the *ebs5 bri1-5* mutant (Figure 2A), which is defective in the Arabidopsis homolog of the yeast Hrd3 and mammalian Sel1L that function as a key recruitment factor to bring a committed ERAD client to the ER membrane anchored E3 ligase (Liu et al., 2011; Su et al., 2011). To eliminate the possibility that the increased bri1-5 abundance in *irb1-1 bri1-5* is caused by increased bri1-5 biosynthesis, we performed a cycloheximide (CHX)-chase experiment, which revealed increased stability of bri1-5 in *irb1-1 bri1-5* compared to *bri1-5* (Figure 2B). Together, these experiments strongly suggested that *irb1-1* partially inhibits ERAD of bri1-5. Based on what were shown in other known Arabidopsis ERAD mutants (Hong et al., 2009, 2012; Su et al., 2011, 2012; Liu et al., 2015), we predicted that increased accumulation of bri1-5 in *irb1-1 bri1-5* would saturate the bri1-5’s ER-retention systems, leading to escape of a small pool of bri1-5 proteins from the ER to the plasma membrane (PM) where bri1-5 could partially activate the BR signaling process. Indeed, a simple biochemical assay using endoglycosidase H (Endo H), an endoglycosidase that removes high-mannose (HM)-type N-glycans of ER-retained glycoproteins but not the complex-type (C-type) N-glycans on proteins that travel through the Golgi body (Faye and Chrispeels, 1985), revealed the presence of a very small pool of bri1-5 proteins carrying the HM-type N-glycan suggestive of ER escape and PM localization (Figure 2A). Contrast to what were previously reported of other ERAD mutations, *irb1-1* was not able to suppress the *bri1-9* mutation (Supplementary Figure 1A). This finding is consistent with the fact that no single *irb1* allele was identified in our previous genetic screens for *bri1-9* suppressors, which led to discoveries of multiple alleles of *EBS1*-EBS7 genes (Jin et al., 2007, 2009; Hong et al., 2009, 2012; Su et al., 2011, 2012; Liu et al., 2015). This is likely caused by weak inhibition of bri1-9 ERAD by the *irb1-1* mutation (Supplementary Figure 1B) combined with a potential weaker receptor function of the surface-localized bri1-9 compared to the PM-localized bri1-5.

### Molecular Cloning of the *IRB1* Gene

**Figure 3:**
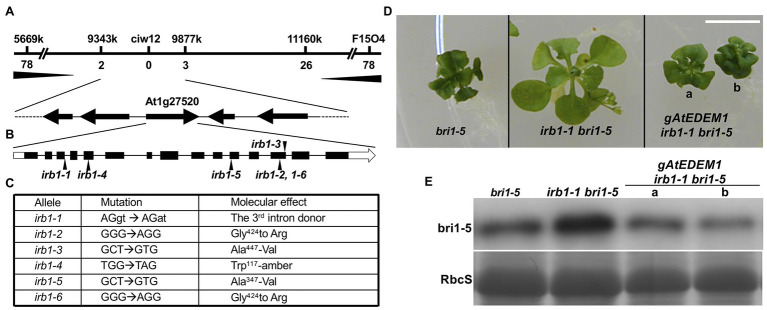
Map-based cloning of the *IRB1* gene. **(A)** The *IRB1* locus was mapped to a ~500-kb genomic region between markers 9,343 and 9,877 k on the top of chromosome I and is closely linked with the marker ciw12 (see Supplementary Table S1 for oligonucleotides of the mapping markers). The horizontal line represents the genomic DNA, and mapping markers and numbers of identified recombinants are shown above and below the line, respectively. **(B)** The gene structure of *At1g27520*. The annotated *At1g27520* gene contains 15 exons (black bar) and 14 introns (thin black line). White boxes denote untranslated regions while black arrows indicate positions of *irb1* mutations. **(C)** Nucleotide changes and molecular defects of 6 *irb1* alleles. **(D)** Four-week-old petri dish-grown seedlings of *bri1-5*, *irb1-1 bri1-5*, and two independent *gAt1g27520*-rescued *irb1-1 bri1-5* transgenic lines. Scale bar = 1 cm. **(E)** Immunoblot analysis of bri1-5 abundance in seedlings shown in **(D)**. Equal amounts of total proteins extracted in 2 X SDS sample buffer from 4-week-old seedlings were separated by 8% SDS–PAGE and analyzed by immunoblots using anti-BRI1 antibody. Coomassie blue staining of the RbcS band of a duplicate gel serves as a loading control.

To understand how the *irb1-1* mutation inhibits ERAD of bri1-5, we cloned the *IRB1* gene using the map-based cloning strategy. The *irb1-1 bri1-5* mutant (in ecotype Ws-2) was crossed with a *bri1-9* mutant (in ecotype Columbia-0 or Col-0) and the resulting F1 plants were allowed to self-fertilization to produce several mapping populations. Genomic DNAs of >1,000 *irb1-1 bri1-5*-like F2 seedlings from these mapping populations were used to determine a close linkage of the *IRB1* locus with an SSLP marker ciw12 (9,621,357–9,621,484, see Supplementary Table S1 for nucleotide sequences) on chromosome I (Figure 3A), which is located close to the Arabidopsis gene *At1g27520* [known previously as MNS5 (Huttner et al., 2014a), 9,558,752–9,563,751] encoding a potential homolog of Htm1/EDEMs that play a key role in the yeast/mammalian ERAD processes (Clerc et al., 2009; Hosokawa et al., 2010; Huttner et al., 2014b). Consistent with the result of our phenotypic analysis of *irb1-1 bri1-9* double mutant, none of these partially suppressed F2 seedlings was homozygous for the *bri1-9* mutation. Sequence analysis of this gene amplified from *bri1-5* and *irb1-1 bri1-5* identified a G-A mutation in *irb1-1 bri1-5* at the third exon/intron junction (AGgt-AGat; Figure 3C), which was predicted to affect the correct splicing of its third intron. RT-PCR analysis of *At1g27520* transcripts with total RNAs isolated from the *irb1-1 bri1-5* and *bri1-5* seedlings identified several aberrantly-spliced *At1g27520* transcripts in *irb1-1 bri1-5* but failed to detect the presence of the correctly-spliced *At1g27520* transcript that could be easily detected in *bri1-5* (Supplementary Figure 2), suggesting that *irb1-1* is likely a null allele of *At1g27520*. The identity of *At1g27520* as *IRB1* was supported by genetic mapping and sequence analysis of five other *irb1* mutants (*irb1-2–irb1-6*; Supplementary Figure 3), each carrying a single nucleotide G-A or C-T mutation in *At1g27520*, which changes Gly^424^ to Arg in *irb1-2* and *irb1-6* (identified in two independent screens), Ala^447^ to Val in *irb1-3*, Trp^117^ to the amber stop codon TAG in *irb1-4*, and Ala^347^ to Val in *irb1-5* (Figure 3C; Supplementary Figure 4). Further support for *At1g27520* being the *IRB1* gene came from a transgenic rescue experiment. As shown in Figures 3C,D, introduction of a 5.3-kb *gAt1g27520* genomic transgene into *irb1-1 bri1-5* not only suppressed its growth phenotype but also reduced its bri1-5 protein abundance.

### IRB1 Is a Homolog of the Yeast Htm1/Mammalian EDEMs and Is Highly Conserved in Land Plants

The *IRB1*/*At1g27520* gene (renamed hereinafter as *AtEDEM1* due to its conserved protein sequence and biochemical function with the mammalian EDEMs) consists of 15 exons and 14 introns (Figure 3B) and encodes a polypeptide of 574 amino acids with a weak signal peptide of 28 amino acids (AAs), which was annotated as one of the two *Arabidopsis* homologs of Htm1/EDEMs recently shown to be functionally redundant in the Arabidopsis ERAD process that degrades both bri1-5 and bri1-9 (Hirao et al., 2006; Quan et al., 2008; Clerc et al., 2009; Hosokawa et al., 2010; Huttner et al., 2014b). IRB1/AtEDEM1 displays 35/54% and 42–47/58–64%, sequence identity and similarity with the yeast Htm1 and three human EDEMs, respectively, within the conserved 430-AA domain of the glycosylhydrolase family 47 (Supplementary Figure 4). The second Arabidopsis Htm1/EDEM homolog, At5g43710 [624 AAs with a longer C-terminal domain, previously known as MNS4 (Huttner et al., 2014b) but was renamed hereinafter as AtEDEM2], exhibits 38/53% and 43–48/61–66% sequence identity and similarity with Htm1 and three human EDEMs, respectively, within its conserved 430-AA domain (Supplementary Figure 4). It should be interesting to note that the sequence identity/similarity between AtEDEM1 and AtEDEM2 are only 47%/61%, which is very similar to the 47/64% and 45/63% sequence identity/similarity between AtEDEM1 and human EDEM1 and between AtEDEM2 and human EDEM2, respectively (Supplementary Figure 4). However, both AtEDEM1 and AtEDEM2 are quite conserved among land plants. AtEDEM1 and AtEDEM2 exhibit 74–98/86–99% and 78–99/87–99% sequence identity/similarity with AtEDEM1 and AtEDEM2 homologs from land plants (Supplementary Figure 5), respectively, including the liverwort *Marchantia Polymorpha* (Bowman et al., 2017), the moss *P. patens* (Rensing et al., 2008), and the spikemoss *Selaginella moellendorffii* (Banks et al., 2011). It is also interesting to note that almost all sequenced land plants contain two EDEM homologs except *Physcomitrella*, which lacks an AtEDEM1 homolog (Supplementary Figure 5) likely due to a gene loss event during its long evolution history.

A direct support for the functional conservation between AtEDEM1 and Htm1/EDEMs came from two reciprocal complementation experiments. A yeast complementation assay showed that the wild-type AtEDEM1 could partially substitute for the yeast Htm1 to stimulate degradation of a yeast model ERAD substrate, an ER-retained mutant variant of the vacuolar carboxypeptidase Y (CPY*) (Ng et al., 2000; Supplementary Figure 6A). Consistently, the *BRI1* promotor-driven expression of the yeast *Htm1* gene could partially suppress the morphological and biochemical phenotype of the *irb1-3 bri1-5* mutant (Supplementary Figure 6B).

### The Two Arabidopsis EDEM Homologs Are Localized in the ER

To investigate if the two Arabidopsis EDEM homologs localize in the ER, we generated C-terminal AtEDEM1/AtEDEM2-GFP fusion transgenes driven by the *BRI1* promoter and transiently expressed the resulting *pBRI1::AtEDEM1/AtEDEM2-GFP* transgene in tobacco (*Nicotiana benthamiana*) leaf epidermal cells along with a known transgene encoding a widely-used ER marker red fluorescent protein tagged with a widely-used ER marker RFP-HDEL (HDEL) ER retrieval motif at its C-terminus [RFP-HDEL; Chakrabarty et al., 2007]. Confocal microscopic examination of the fluorescent patterns of agro-infiltrated tobacco leaves revealed that the green fluorescent patterns of the two AtEDEM-GFPs overlapped nicely with that of RFP-HDEL (Supplementary Figure 7A), indicating that both AtEDEM1 and AtEDEM2 are localized in the ER.

Consistent with our microscopic results, Endo H-analysis of the two transiently-expressed GFP-fusion proteins showed that both AtEDEMs were Endo-H sensitive (Supplementary Figure 7B) and were thus glycosylated with HM-type N-glycans indicative of ER-localization. By contrast, the GFP-tagged At1g30000 (a predicted ER α1,2-mannosidase I homolog, also known as MNS3; Liebminger et al., 2009) and GFP-tagged At1g51590 (one of the two Golgi-type α1,2-mannosidases, also known as MNS1; Liebminger et al., 2009) were found to be Endo-H resistant despite being predicted to carry 5 and 3 N-glycosylation sites, respectively (Supplementary Figure 7B). Our results on the cellular localization of At1g30000/MNS3, At1g51590/MNS1, and the two AtEDEMs are consistent with three published studies on the five Arabidopsis α1,2-mannosidases (Liebminger et al., 2009; Huttner et al., 2014b; Schoberer et al., 2019).

### The α1,2-Mannosidase Activity Is Required for the Biological Function of AtEDEM1

**Figure 4:**
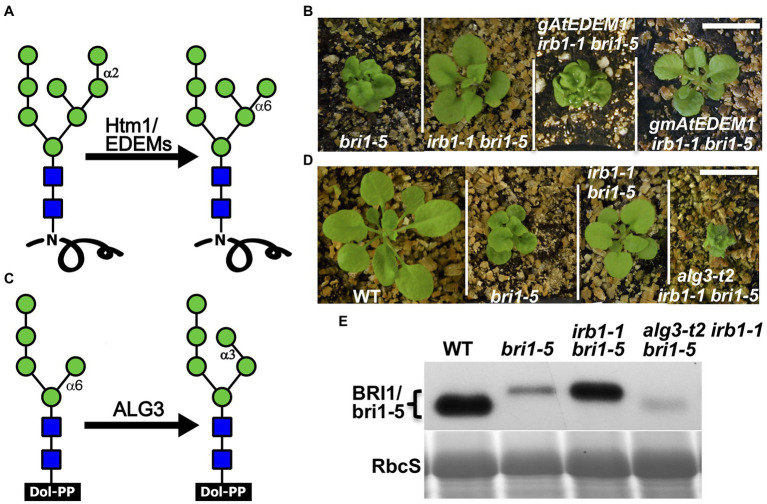
AtEDEM1 promotes bri1-5 ERAD likely through its predicted α1,2-mannosidase activity. **(A)** Schematic presentation of the α1,2-mannosidase activity of Htm1/EDEMs. **(B)** Photographs of 4-week-old soil-grown plants of *bri1-5*, *irb1-1 bri1-5*, *gAtEDEM1 irb1-1 bri1-5*, and *gmAtEDEM1 irb1-1 bri1-5* transgenic lines. **(C)** A schematic presentation of the mannosyltransferase activity of Asparagine-Linked Glycosylation 3 (ALG3). **(D)** Photographs of 4-week-old soil-grown plants of WT, *bri1-5*, *irb1-1 bri1-5*, and *alg3-t2 irb1-1 bri1-5* mutants. In **(B,D)**, scale bar = 1 cm. **(E)** Immunoblotting analysis of the bri1-5 abundance. Equal amounts of total proteins extracted in 2X SDS buffer from 4-week-old leaves were separated by 8% SDS-PAGE and analyzed by immunoblot with anti-BRI1 antibody. Coomassie blue staining of RbcS on a duplicated gel serves loading control.

Our previous study demonstrated that ERAD of both bri1-5 and bri1-9 requires the conserved α1,6-Man-exposed N-glycan signal (Hong et al., 2012), which was known to be generated in yeast and mammals by the α1,2-mannosidase activity of Htm1/EDEMs (Figure 4A; Quan et al., 2008; Clerc et al., 2009; Hosokawa et al., 2010). Interestingly, an earlier study suggested that the mannosidase activity of EDEM1 might not be important to promote ERAD in cultured mammalian cells as several catalytically-dead EDEM1 proteins could enhance degradation of known ERAD substrates (Cormier et al., 2009; Ninagawa et al., 2014). To investigate if AtEDEM1 absolutely requires its predicted α1,2-mannosidase activity for its role in ERAD or has an α1,2-mannosidase-independent function in promoting bri1-5 degradation, we performed a PCR-based site-directed mutagenesis experiment with the *irb1-1*-complementing *gAtEDEM1* genomic construct to mutate Glu^134^ (corresponding to the human EDEM1’s Glu^220^ known to be essential for its α1,2 mannosidase activity; Hosokawa et al., 2010) to glutamine (Q), and transformed the resulting mutant transgene into the *irb1-1 bri1-5* double mutant. As shown in Figure 4B, the E^135^-Q-mutated *gmAtEDEM1* (m indicating mutant) transgene failed to complement the *irb1-1* mutation in the *bri1-5* background, indicating that the function of AtEDEM1 in promoting bri1-5 ERAD absolutely requires its predicted α1,2-mannosidase activity.

### The *alg3* Mutation Could Nullify the Suppressive Effect of the *irb1* Mutation on *bri1-5* Dwarfism

Earlier studies indicated that the other α1,6-Man residue on N-linked glycans of misfolded proteins (Figure 4C), when being exposed, could also function as an ERAD N-glycan signal that can be recognized and bound by the ERAD receptor Yos9/OS-9/EBS6 (Quan et al., 2008; Clerc et al., 2009; Hong et al., 2012). We reasoned that if the effect of the *irb1* mutations on ERAD of bri1-5 was indeed caused by a failure or a reduced rate of generation of the conserved ERAD N-glycan signal carrying a free α1,6-Man residue, the suppressive effects of the *irb1-1* mutation on the *bri1-5* dwarfism and bri1-5 ERAD would be eliminated by a loss-of-function mutation of Asparagine-Linked Glycosylation 3 (ALG3), a highly-specific mannosyltransferase that adds an α1,3-Man residue to the other α1,6-Man residue (Henquet et al., 2008; Kajiura et al., 2010). Loss-of-function *alg3* mutations result in N-glycosylation of glycoproteins with a truncated Man_5_GlcNAc_2_ glycan carring a different free α1,6-Man residue (Figure 4C). Indeed, when crossed into the *irb1-1 bri1-5* double mutant, a T-DNA insertional *alg3* mutation, *alg3-t2* that was previously reported (Hong et al., 2012), nullified the suppressive effect of the *irb1-1* mutation on *bri1-5*. As shown in Figure 4D, the *alg3-t2 irb1-1 bri1-5* triple mutant is a much severe dwarf mutant than the *bri1-5* mutant. Consistent with the enhanced dwarfism phenotype, immunoblot assay showed that the *alg3-t2* mutation not only increased the mobility of the bri1-5 band (due to smaller N-glycans) but also reduced the bri1-5 protein level below that of the *bri1-5* single mutant (Figure 4E). We thus concluded that the inhibition of bri1-5 degradation in the *irb1-1 bri1-5* double mutant is caused by inhibition of generating the α1,6-Man-exposed N-glycans on the mutant BR receptor.

### AtEDEM1 and AtEDEM2 Play a Redundant Role in ERAD of bri1-5

Our finding that the *irb1-1* mutant is a weak ERAD mutant of bri1-5 coupled with the fact that the Arabidopsis genome encodes two potential EDEM homologs (Huttner et al., 2014b) prompted us to test the possibility that AtEDEM1 functions redundantly with AtEDEM2 in degrading bri1-5. To test our hypothesis, we first transformed a genomic *gAtEDEM2* transgene into the *irb1-1 bri1-5* mutant and found that while this transgene was able to rescue the *irb1-1* mutation, the percentage of rescued *irb1-1 bri1-5* plants among the resulting *gAtEDEM2 irb1-1 bri1-5* transgenic lines (a total of 70 lines) was relatively low (~20%; Supplementary Figure 8A) compared to 90% of rescued *gAtEDEM2 irb1-1 bri1-5* lines (out of 58 lines). This difference in the *irb1-1*-rescuing activity could be caused by the weaker promoter or weaker catalytic activity of AtEDEM2. To differentiate these two possibilities, we created two additional transgenic constructs *pBRI1::cAtEDEM1* and *pBRI1::cAtEDEM2* (c stands for cDNA) using the *BRI1* promoter (*pBRI1*) to drive the expression of *AtEDEM1* or *AtEDEM2*, transformed each transgene into the *irb1-1 bri1-5* double mutant, and analyzed the resulting transgenic plants. The transgenic expression of each cDNA construct not only complemented the *irb1-1* mutation but also led to severe dwarfism compared to the parental *bri1-5* mutant, although the percentage of severely dwarfed transgenic lines is higher with the *pBRI1::cAtEDEM1* transgene than with the *pBRI1::cAtEDEM2* transgene (Supplementary Figure 8B). Together, these results suggested that the weaker physiological activity of AtEDEM2 in bri1-5 ERAD is likely contributed by its weaker promoter and its weaker biochemical activity.

**Figure 5:**
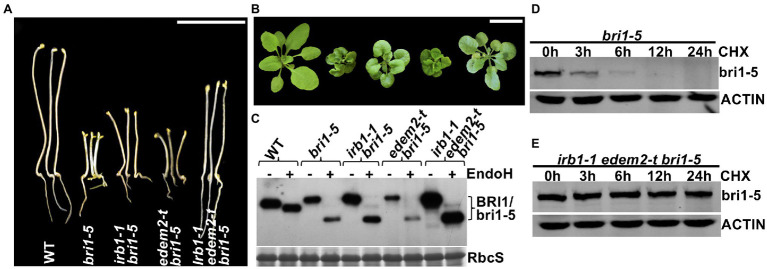
AtEDEM1 and AtEDEM2 function redundantly in promoting bri1-5 ERAD. **(A)** Pictures of 5-day-old dark-grown seedlings of WT, *bri1-5*, *irb1-1 bri1-5*, *edem2-t bri1-5*, and *irb1-1 edem2-t bri1-5*. **(B)** Photographs of 6-week-old soil-grown seedlings. In **(A,B)**, scale bar = 1 cm. **(C)** Immunoblot analysis of the bri1-5 abundance. Equal amounts of total proteins extracted from 2-week-old light-grown seedlings were treated with or without Endo H, separated by 8% SDS–PAGE, and analyzed by immunoblotting using an anti-BRI1 antibody. The lower strip is Coomassie blue-stained RbcS bands of a duplicated gel as a loading control. **(D–E)** Immunoblotting analysis of the bri1-5 stability in *bri1-5* and *irb1-1 edem2-t bri1-5* mutant seedlings. Two-week-old seedlings were carefully transferred into liquid ½ MS medium containing 180 μM CHX for continued growth. Equal amounts of seedlings were taken out at different time points and were immediately used to extract total proteins with 2 X SDS sample buffer. The proteins were subsequently separated by 8% SDS-PAGE and analyzed by western blot with anti-BRI1 antibody. The same filters were also probed with anti-ACTIN antibody to control for equal sample loading.

The functional redundancy between AtEDEM1 and AtEDEM2 was further supported by our genetic study. We obtained a T-DNA insertional mutant for AtEDEM2 (*SALK_095857*, named hereinafter as *edem2-t*) and crossed the mutation into *bri1-5*. RT-PCR analysis showed that the T-DNA insertion resulted in no detectable level of the *AtEDEM2* transcript (Supplementary Figure 9) while phenotypic examination indicated that the *edem2-t* mutation was not able to suppress the dwarf phenotype of dark or light-grown *bri1-5* mutant or to inhibit the bri1-5 ERAD (Figures 5A–C), explaining why several independent genetic screens for *bri1-5* suppressors failed to uncover a single *edem2* mutation. However, when the *edem2-t* mutation was crossed into the *irb1-1 bri1-5* double mutant, it enhanced the suppressive effect of the *irb1-1* mutation on *bri1-5*. As shown in Figures 5A,B, the triple *irb1-1 edem2-t bri1-5* mutant has a longer hypocotyl in the dark and is noticeably larger in the light than the *irb1-1 bri1-5* double mutant. Consistent with the morphological phenotypes, the abundance of bri1-5 in *irb1-1 edem2-t bri1-5* is significantly higher than that of the *irb1-1 bri1-5* double mutant (Figure 5C). A CHX-chase experiment indicated that the increased abundance of bri1-5 in the *irb1-1 edem2-t bri1-5* triple mutant was caused by near complete inhibition of bri1-5 degradation rather than by increased protein synthesis (Figures 5D,E). More importantly, the amount of the Endo H-resistant form of bri1-5, which was thought to be localized on the PM (Hong et al., 2008), is also higher in the triple mutant than the *irb1-1 bri1-5* mutant (Figure 5C). Taken together, these results demonstrated that AtDEDM1 and AtEDEM2 function redundantly in the ERAD process that degrades bri1-5, which is consistent with an earlier study on the physiological functions of AtEDEM1 and AtEDEM2 (Huttner et al., 2014b). More importantly, our study revealed that AtEDEM1 exhibits a stronger physiological activity in promoting bri1-5 degradation due to its stronger promoter and a stronger biochemical activity.

### Simultaneous Elimination of AtEDEM1 and AtEDEM2 Results in Inhibition of the C-Branch Terminal α1,2-Man-Residue Trimming

To directly examine the impact of simultaneous elimination of the two EDEM homologs on the α1,2-Man residue-trimming activity on misfolded glycoproteins, we intended to analyze the N-glycans on bri1-5 in Arabidopsis mutants. Due to the failure of the anti-BRI1 antibody to immunoprecipitate the endogenous BRI1/bri1-5 protein, we generated a *pBRI1::bri1-5ED-GFP-HDEL* transgene, consisting of the 1.5-kb *BRI1* promoter, the coding sequences of the entire extracellular domain (ED) of bri1-5 and green fluorescent protein tagged with the HDEL ER-retrieval motif (Supplementary Figure 10A), transformed it into the wild-type, an *irb1-1 edem2-t* double mutant, and an *ebs5-1* mutant. The last mutant is defective in a key client-recruitment factor that recognizes and brings an ERAD substrate to the ER membrane anchored E3 ligase (Su et al., 2011) and was used to stabilize the bri1-5ED-GFP-HDEL fusion protein for easy detection of exposed α1,6-Man residue. An Endo H-immunoblot assay with the total proteins of the resulting *pBRI1::bri1-5ED-GFP-HDEL* (in the wild-type background) transgenic lines showed that the engineered bri1-5ED-GFP-HDEL was indeed retained in the ER (Supplementary Figure 10B). As expected, a Kif treatment experiment revealed that bri1-5ED-GFP-HDEL was degraded *via* a glycan-dependent manner (Supplementary Figure 10C), indicating that bri1-5ED-GFP-HDEL could be used as a reporter to analyze the impact of the double mutation of AtEDEM1 and AtEDEM2 on the α1,2-Man-trimming reactions of an ERAD client in Arabidopsis.

**Figure 6:**
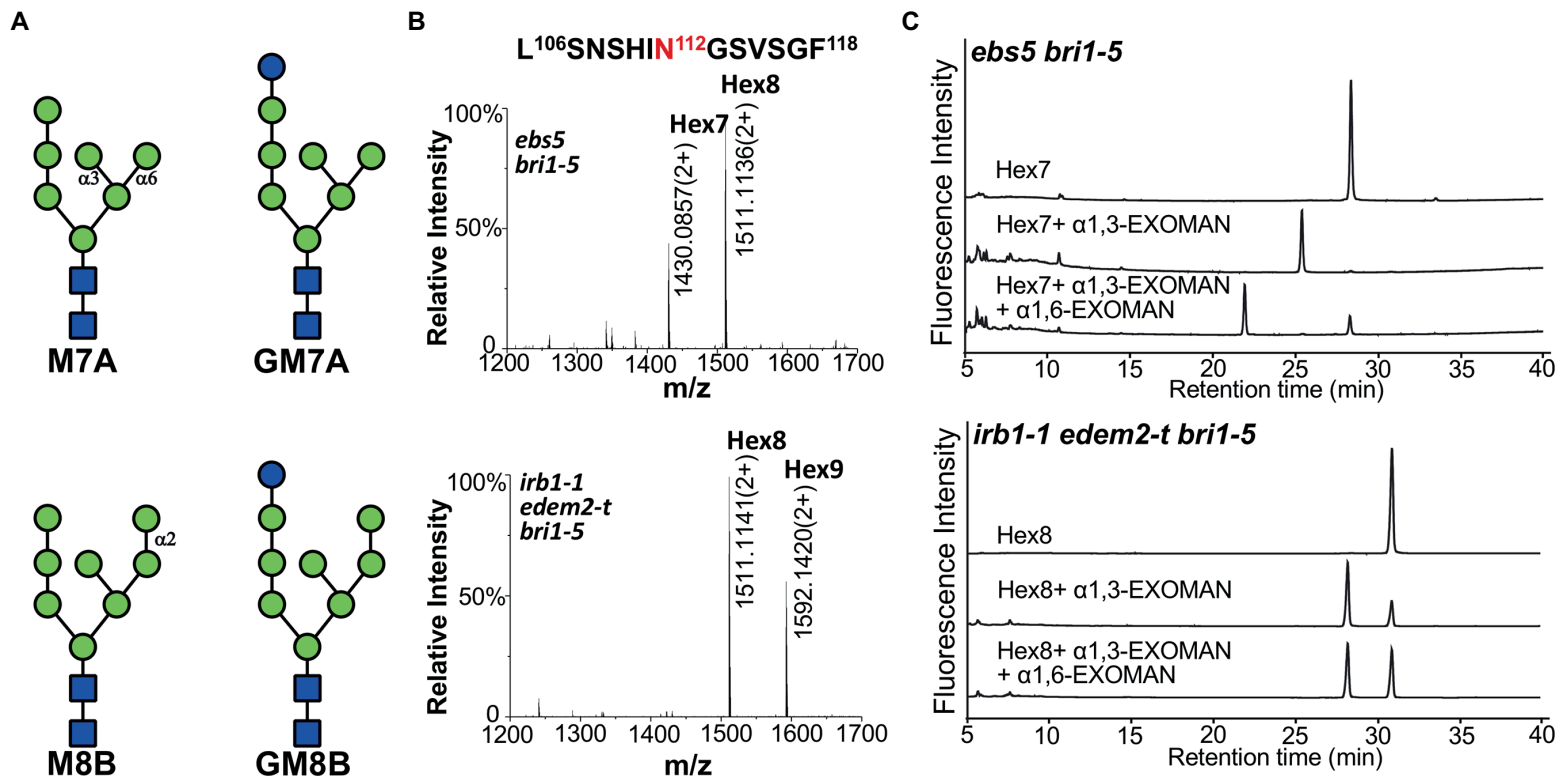
The double mutation of AtEDEM1 and AtEDEM2 block the C-branch terminal α1,2-Man trimming. **(A)** Structural features of two different high-mannose N-glycans. The N-glycan structures were determined by Orbitrap Fusion LC–MS/MS on the chymotrypsin-cleaved peptides of the GFP-tagged bri1-5ED fusion proteins, which were immunoprecipitated from *ebs5 bri1-5* (two structures in the upper panel) and *irb1-1 edem2-t bri1-5* (two structures in the bottom panel) mutant seedlings. **(B)** Mass spectrometric profiles of the high abundance glycopeptides from the chymotrypsin digest of the immunoprecipitated bri1-5ED-GFP-HDEL in the *ebs5 bri1-5* (upper panel) and *irb1-1 edem2-t bri1-5* (lower panel). The doubly charged ions, as shown in each spectrum, correspond to two different high mannose N-glycans linked to residue Asn^112^ of the peptide sequence LASNSHI-N^112^GSVSGF, respectively. **(C)** Ultra-performance liquid chromatography (UPLC)-based analysis of α-mannosidase cleaved products of the purified N-glycans of bri1-5ED-GFP-HDEL immunoprecipitated from *ebs5* and *irb1-1 edem2-t bri1-5* mutants.

A representative transgenic line in each mutant background was used to immunoprecipitate bri1-5ED-GFP-HDEL, which was subsequently analyzed by high-resolution liquid chromatography tandem mass spectrometry (LC–MS/MS) to determine the N-glycan structures of the ER retained fusion protein. As shown in Figures 6A,B, two major N-glycans, Hex_7_GlcNAc_2_ (i.e., Man7; Hex refers hexose) and Hex_8_GlcNAc_2_ (i.e., GlcMan7; Glc refers glucose) were detected at the glycosylation site at residue Asn^112^ position of the chymotrypsin-digested bri1-5ED-GFP-HDEL peptide LSNSHIN^112^GSVSGF in the *ebs5* mutant. In contrast, a different N-glycan profile was observed in the mass spectrum of the protein digest in the *irb1-1 edem2-t* double mutant, in which the two glycopeptide ions were identified to contain N-glycans of Hex_8_GlcNAc_2_ (i.e., Man8) and Hex_9_GlcNAc_2_ (i.e., GluMan8). Similar N-glycan distributions were also observed at other glycosylation sites, for example, the two distinct N-glycans were presented at residue Asn^636^ of peptide NPCN^636^ITSR of the trypsin digest of the immunoprecipitated bri1-5ED-GFP-HDEL (Supplementary Figure 11). The observation of the difference of one Man residue in the N-glycan structures at residues Asn^112^ and Asn^636^ of bri1-5ED-GFP-HDEL between *ebs5* and the *irb1-1 edem2-t* double mutant is consistent with the functional conservation between AtEDEM1 and Htm1, strongly suggesting that the *irb1-1 edem2-t* double mutations likely inhibit the Man-trimming activity essential for ERAD of bri1-5.

To validate the exact position of the AtEDEM1/AtEDEM2-trimmed α1,2-Man residue in the N-glycan structures, the immunoprecipitated bri1-5ED-GFP-HDEL fusion protein was treated with PNGase F, an amidase that cleaves the covalent bond between the innermost GlcNAc residue and the glycosylated Asn residue (Tarentino et al., 1985). The released N-glycans were fluorescently labeled with 2AB and subsequently separated by HILIC. Individual N-glycan fractions were collected, digested with specific α1,3-, α1,6-, or α1,2/3/6-exomannosidases, and analyzed by UPLC. Figure 6C; Supplementary Figure 12 show that Hex_7_GlcNAc_2_ and Hex_8_GlcNAc_2_ glycans accumulated in *ebs5* were sensitive to both α1,3- and α1,6-mannosidases, indicating that Hex_7_GlcNAc_2_ is Man_7_GlcNAc_2_ with free α1,3-Man and α1,6-Man residues (Figure 6A). The same cleavage response of Hex_8_GlcNAc_2_ to both α-mannosidases (Supplementary Figure 12) indicated that this is a monoglucosylated GlcMan_7_GlcNAc_2_ glycan, which is consistent with our earlier conclusion that bri1-5 is retained in the ER by several independent retention mechanisms that include the UGGT-CRT/CNX system (Hong et al., 2008). Our α-mannosidase analysis of the PNGase F-cleaved N-glycans of the immunoprecipitated bri1-5ED-GFP-HDEL of the *irb1-1 edem2-t bri1-5* triple mutant showed that the Hex_8_GlcNAc_2_ glycan was sensitive only to the α1,3-exomannosidase but could not be cleaved by the α1,6-exomannosidase (Figure 6C), indicating the presence of a terminal α1,2-Man residue that protects the α1,6-Man residue from its cleavage by the α1,6-exomannosidase. Taken together, these biochemical analyses confirmed that the *irb1 edem2-t* double mutation blocks the removal of the C-branch α1,2-Man residue.

### The Arabidopsis EDEMs Catalyze a Rate-Limiting Step of a Plant ERAD Pathway

**Figure 7:**
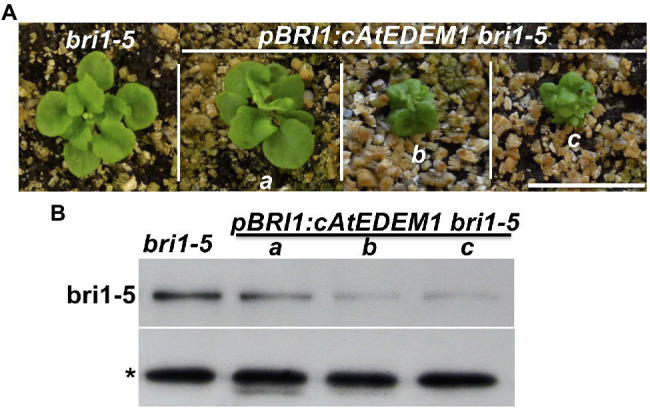
Overexpression of AtEDEM1 stimulates bri1-5 degradation. **(A)** Photographs of 4-week-old soil-grown plants of *bri1-5* and *pBRI1::AtEDEM1 bri1-5* transgenic lines. Scale bar = 1 cm. **(B)** Immunoblot analysis of the bri1-5 abundance in plants shown in **(A)**. The star sign indicates a non-specific cross-reacting band used as a loading control.

While performing the transgenic rescue experiments, we noticed that some of the *gAtEDEM1* transgenic lines exhibited stronger dwarfism than the parental *bri1-5* strain (Figure 3C). Similar stronger dwarf phenotypes were not observed in our previous experiments that overexpressed *EBS5* or *EBS6* in the *bri1-5* mutant, whose protein products work together to bring a committed ERAD client to the membrane-anchored Hrd1 E3 ligase complex (Su et al., 2011, 2012), suggesting that recognition and recruitment of a marked bri1-5 to the ERAD machinery is not the rate-limiting step. By contrast, similar severe dwarfism phenotypes were observed in the *alg3 ebs3 bri1-9* or *alg3 irb1-1 bri1-5* triple mutant containing mutant BR receptors glycosylated with α1,6-Man-exposing N-glycans that mark a misfolded protein for ERAD (Hong et al., 2012; Figure 4D), indicating that generating the ERAD N-glycan signal is a major rate-limiting step of the Arabidopsis ERAD pathway. Consistent with the severe dwarfism phenotype, an immunoblot analysis showed that the abundance of bri1-5 in those severely-dwarfed *gAtEDEM1 irb1-1 bri1-5* transgenic lines or *alg3 irb1-1 bri1-5* triple mutant was much lower than that of the parental *bri1-5* mutant (Figures 3D, 4D). Severely dwarfed transgenic *irb1-1 bri1-5* mutants were also observed when the *BRI1* promoter was used to drive overexpression of the cDNA transgene of *AtEDEM1* or *AtEDEM2* in the *irb1-1 bri1-5* mutant (Supplementary Figures 6B,C). When transformed into the single *bri1-5* mutant, the *pBRI1::AtEDEM1* transgene also caused severe dwarfism in the resulting *pBRI1::AtEDEM1 bri1-5* transgenic lines and immunoblot analysis showed that the severely-dwarfed transgenic lines accumulated less bri1-5 than the parental *bri1-5* mutant (Figures 7A,B). Taken together, our data strongly suggested that the AtEDEM1/AtEDEM2-mediated cleavage of the C-branch terminal α1,2-Man residue is a major rate-liming step in the Arabidopsis ERAD pathway that degrades ER-retained mutant bri1-5 receptor.

## Discussion

### A Predominant Role of AtEDEM1 in the ERAD Pathway That Degrades bri1-5

While an earlier study using the reverse genetic approach showed that neither *edem1/mns5* nor *edem2/mns4* but an *mns4 mns5* double mutation had any visible impact on the dwarf phenotypes of *bri1-5* and *bri1-9* and concluded that the two Arabidopsis Htm1/EDEM homologs function redundantly in promoting degradation of bri1-5 and bri1-9 (Huttner et al., 2014b), our forward genetic study revealed that six EMS-introduced single nucleotide changes in AtEDEM1 partially suppress the *bri1-5* dwarfism by weakly inhibiting bri1-5 degradation. Interestingly, the null *irb1-1* mutation (due to defective splicing-caused early translational termination) failed to suppress the *bri1-9* mutation, which is likely attributed to its weak inhibition of the ERAD pathway that degrades bri1-5 and bri1-9 and a potentially weaker receptor function of the PM-localized bri1-9 compared to bri1-5. Consistent with the earlier study (Huttner et al., 2014b), a loss-of-function *edem2*-*t* mutation had no effect on bri1-5 degradation and no single EMS-introduced *edem2* mutation has so far been uncovered in several forward genetic screens for extragenic suppressors of *bri1-5* and *bri1-9* mutants, which discovered multiple alleles of *EBS1-EBS7* genes, revealing potential difference between the two Arabidopsis EDEM homologs in stimulating ERAD of bri1-5 and bri1-9. Consistently, while a genomic *gAtEDEM1* transgene could achieve >90% phenotypic complementation out of 83 *gAtEDEM1 irb1-1 bri1-5* transgenic mutants, a similar genomic *gAtEDEM2* transgene only led to ~20% of the *gAtEDEM2 irb1-1 bri1-5* transgenic lines being phenotypically similar to or severer than *bri1-5*. Importantly, when driven by the same *pBRI1* promoter, AtEDEM1 produced higher percentage of severely dwarfed transgenic mutant than AtEDEM2 in the *irb1-1 bri1-5* mutant. As expected, a T-DNA insertional mutation in AtEDEM2 (*edem2*-*t*) mutation significantly enhances the suppressive effect of *irb1-1* on the *bri1-5* dwarfism as simultaneous elimination of the two AtEDEMs completely blocks the ERAD of bri1-5, leading to markedly increased amount of ER-escaping bri1-5 carrying the C-type N-glycans. Thus, our study demonstrated that despite functional redundancy of AtEDEM1/MNS5 and AtEDEM2/MNS4, the former α1,2-mannosidase plays a predominant role in an ERAD process that degrades bri1-5. Our conclusion is supported by a recent study revealing a non-redundant role of AtEDEM1/MNS5 in the Arabidopsis ERAD pathway (Sun et al., 2022).

Our study provided further genetic and biochemical support for the key role of the two AtEDEMs in generating the conserved N-glycan signal to mark ERAD substrates. First, both AtEDEMs exhibit significant sequence identity/similarity with Htm1/EDEMs, and AtEDEM1 and Htm1 complemented each other’s loss-of-function mutation (Supplementary Figure 6). Second, a catalytically-dead mutant of AtEDEM1 failed to rescue the *irb1-1* mutation while the wild-type copy of the transgene fully complemented the *irb1-1* mutation, implying that the predicted α1,2-mannosidase activity is essential for its physiological function in degrading the mutant bri1-5 receptor. Third, a T-DNA insertional *alg3-t* mutation, which causes N-glycosylation with truncated Man_5_GlcNAc_2_ glycans carrying a different free α1,6-Man residue, could nullify the suppressive effect of the *irb1-1* mutation on the *bri1-5* dwarfism and its inhibitory effect on the ERAD of bri1-5, implying that the *irb1-1* mutant was compromised in the activity to generate α1,6-Man-exposed N-glycans on misfolded glycoproteins. Finally, N-glycan analysis of GFP-tagged bri1-5ED proteins purified from *bri1-5* and *irb1-1 edem2 bri1-5* mutant provided a direct *in vivo* evidence for the two AtEDEMs being the ER-localized C-branch-specific α1,2-mannosidases. However, it remains a possibility that AtEDEMs might function as the necessary cofactors for the suspected C-branch α1,2-mannosidases. Therefore, *in vitro* enzyme assays using heterologouslly-expressed AtEDEMs will be needed to definitively prove that AtEDEM1 and AtEDEM2 are indeed active α1,2-mannosidases that catalyze the C-branch α1,2-Man trimming reaction of the ER-retained misfolded glycoproteins.

In addition, our investigation revealed that the generation of the conserved N-glycan signal constitute a rate-limiting step in the Arabidopsis ERAD process that degrades bri1-5. Our earlier studies showed that overexpression of EBS5 or EBS6, which work together to recruit a committed ERAD client to the ER membrane-anchored Hrd1 E3 ligase, failed to enhance the dwarfism of *bri1-5* and/or *bri1-9* and to stimulate degradation of the corresponding mutant BR receptors (Su et al., 2011, 2012). In this study, we showed that overexpression of AtEDEM1 or AtEDEM2 could enhance the *bri1-5* dwarf phenotype and stimulate bri1-5 degradation, which is consistent with the morphological and biochemical phenotypes of *alg3 irb1-1 bri1-5* or *alg3 ebs3 bri1-5/bri1-9* mutants that have their ER-localized glycoproteins to be decorated with N-glycans carrying another exposed α1,6-Man residue (Hong et al., 2012). Our revelation is also consistent with studies in yeast and mammalian systems, which suggested that the α1,2-Man-trimming reaction (that drives a misfolded glycoprotein into the ERAD pathway) is a slow process to favor refolding over removal.

### The Two Arabidopsis EDEMs Evolved Independently in the Green Lineage and May Participate in Different Physiological Processes

Although the two AtEDEMs exhibit relatively low sequence homology (50% identity/64% similarity) with each other, each AtEDEM displays 71–99% identity/81–99% similarity with their respective orthologs from other land plant species, suggesting a very ancient gene duplication event that generated the two EDEM paralogs in the green lineage. It is interesting to note that at least three detected *irb1* mutations, *irb1-2/irb1-6* changing Gly^424^ to Arg and *irb1-5* mutating Ala^347^ to Val, alter amino acids that are only conserved in EDEM1 as Gly^424^ is replaced by Cys and Ala^347^ is replaced by Pro in EDEM2s (Supplementary Figure 4). BLAST searches against existing databases indicated that the genome of the earliest land plant *M. polymorpha* (Bowman et al., 2017) encodes homologs of both AtEDEMs whereas the *Physcomitrella* genome only encodes an AtEDEM2 homolog (Rensing et al., 2008). Similarly, the sequenced genomes of two charophyte green algae whose ancestor was thought to give rise to land plants showed that while *Klebsormidium flaccidum* has homologs of both AtEDEMs (Hori et al., 2014), *Chara braunil*, which is more closely-related to land plants, has only an AtEDEM2 homolog (Nishiyama et al., 2018; Supplementary Figure 5). We suspect that the *AtEDEM1* homologous gene might be lost in the genomes of the moss and the *Charophyceae* alga during their 400–500 million year-evolution history. It is interesting to note that a dozen of recently-sequenced *Chlorophyte* algae genomes encode no, one, or two homologs of the AtEDEMs (Supplementary Figure 5). For example, *M. pusilla* RCC299, *M. pusilla* CCMP1545, and *Bathycoccus parasinos* (all in the Prasinophytes family) contain a potential AtEDEM1 homolog while the genomes of *Ostreococcus lucimarinus CCE9901*, *Ostreococcus tauri*, and *Chlorella variabilis* encode a potential AtEDEM2 homolog. Interestingly, the three fully-sequenced *Chlorophycean* green algae, *Chlamydomonas reinhardtii, Dunaliella salina* CCAP19/18 and *Volvox carteri*, lack any AtEDEM homolog, but *Coccomyxa subellipsoidea* C-169 (known previously as *Chlorella vulgaris* and a close relative of *Chlorella variabilis* NC64A), which belongs to the class *Trebouxiophyceae*, contains homologs of both AtEDEMs (Supplementary Figure 5). These analyses strongly suggest that the gene duplication event that created the two EDEMs in the green lineage occurred before the splitting of Streptophytes (consisting of the land plants and their closely-related green algae such as *K. flaccidum* and *C. braunii*) and Chlorophytes (containing most of the remaining green algae). Further phylogenetic studies are needed to know if the EDEM1/2 gene duplication predated the animal–plant split.

Both plant EDEMs are highly conserved throughout the long evolution of the green lineage, implying that each EDEM plays distinct evolutionarily-conserved physiological functions despite shared biochemical activity. As discussed above, our transgenic experiments with *gAtEDEM1/2* genomic and *pBRI1::AtEDEM1/2* cDNA transgenes revealed that the two Arabidopsis EDEMs exhibited a clear difference in rescuing the null *irb1-1* mutation, which is likely due to different promoter activities and potential difference in biochemical activities of the two AtEDEMs. Gene expression analysis of the two AtEDEMs using the Arabidopsis eFP browser 2.0 (http://bar.utoronto.ca/efp2/Arabidopsis/Arabidopsis_eFPBrowser2.html; Winter et al., 2007) not only revealed a largely-overlapping expression pattern but also detected tissues where *IRB1/AtEDEM1* or *AtEDEM2* is expressed higher than the other (Supplementary Figure 13), suggesting their involvement in different developmental and physiological processes. Analysis of gene co-expression profiles using ATTED-II (Obayashi et al., 2009) seems to support our hypothesis. As shown in Supplementary Figure 14, it is *AtEDEM2* but not *AtEDEM1* that is co-expressed with known and/or predicted ER chaperones/folding catalysts. A similar finding was previously reported for three Arabidopsis CRTs (Jin et al., 2009). While the two highly conserved Arabidopsis CRTs, CRT1, and CRT2, were known to be co-expressed with ER chaperones/folding enzymes, the plant-specific CRT3, which was responsible for retaining bri1-9 in the ER, was shown to be coexpressed with genes implicated in plant stress tolerance (Jin et al., 2009). Detailed phenotypic analysis with the *irb1*, *edem2-t*, and *irb1 edem2-t* mutants or transgenic lines that overexpress AtEDEM1 or AtEDEM2 could be used to investigate if the two AtEDEMs have overlapping yet distinctive biological functions during plant growth and development or plant stress tolerance.

### Is EDEM a Folding Sensor of the ERAD Pathway?

One of the remaining mysteries of the ERAD process is how the system determines if a nonnative glycoprotein is a folding intermediate, a repairable or irreparable misfolded protein and should thus be allowed to continue its folding/refolding process or be condemned into the ERAD process. It was previously suggested that the yeast MNS1, the ER-localized α1,2-mannosidase that specifically cleaves the terminal α1,2-Man residue from the middle branch of N-linked Man_9_GlcNAc_2_ glycan, serves as a timer, due to its slow enzymatic kinetics, to create a discrete time window for a given glycoprotein to attain its native conformation before being marked for degradation by ERAD (Su et al., 1993; Helenius et al., 1997). Although recent studies have convincingly shown that it is Htm1 and EDEMs that generate the conserved N-glycan ERAD signal, the MNS1/ERManI-mediated middle branch α1,2-Man trimming remains a key event for ERAD because Htm1/EDEMs act only on B-branch trimmed Man_8_GlcNAc_2_ but not untrimmed Man_9_GlcNAc_2_ for removing the C-branch terminal α1,2-Man residue (Quan et al., 2008; Clerc et al., 2009). Thus, MNS1/ERManI could still be functionally involved in differentiating a terminally-misfolded glycoprotein from reparable misfolded protein or a folding intermediate, especially when considering a recent *in vitro* assay showing that the human ERManI preferentially removes α1,2-Man residues from unfolded/misfolded glycoproteins (Aikawa et al., 2012, 2014). However, recent studies demonstrated that both animal and plant homologs of the yeast MNS1 are not localized in the ER but were instead found mainly in the Golgi body or the ER-Golgi intermediate compartment, making ERManI less likely to be the folding sensor of the ERAD pathway (Huttner et al., 2014b; Benyair et al., 2015; Schoberer et al., 2019). More importantly, a recent study showed that a T-DNA insertion of the Arabidopsis homolog of MNS1/ERManI fails to suppress the dwarf phenotype of *bri1-9* and *bri1-5*, suggesting that the generation of a conserved N-glycan ERAD in plants might not require the B-branch α1,2-Man-trimming step (Huttner et al., 2014b). It is worthy to mention that a recent yeast study did uncover a Htm1-dependent but MNS1-independent ERAD pathway (Hosomi et al., 2010).

Given their crucial roles in generating the necessary ERAD N-glycan signal and their ER location, Htm1/EDEMs could be directly involved in differentiating terminally misfolded glycoproteins from reparable misfolded glycoproteins or folding intermediates (Shenkman et al., 2018). EDEMs were previously shown to function as molecular chaperones that can bind non−/misfolded proteins but not their native conformers (Hosokawa et al., 2006). However, this chaperone function alone will not qualify Htm1/EDEMs as the folding sensor capable of differentiating a terminally-misfolded protein from reparable misfolded proteins or folding intermediates because all these proteins have hydrophobic residue-exposing surfaces that would interact with a molecular chaperone. EDEMs might need a partner to function as an ERAD folding sensor. Indeed, several recent studies suggested that a protein disulfide isomerase (PDI) might be such a factor that works together with EDEMs to preferentially act on terminally-misfolded glycoproteins that are trapped into a non-native folding state (Gauss et al., 2011; Pfeiffer et al., 2016; Liu et al., 2016b). Similarly, three recent studies have shown that binding of ERdj5, ERp56, or TXNDC11 (thioredoxin domain containing 11), three members of the mammalian PDI family (Kozlov et al., 2010), was required to stimulate the redox-sensitive α1,2-Man-trimming activity of EDEM1 and EDEM3 (Timms et al., 2016; Lamriben et al., 2018; Shenkman et al., 2018; Yu et al., 2018). Direct testing AtEDEM2-PDI interaction or identifying AtEDEM-binding proteins could lead to a better understanding on how the two AtEDEMs select their substrates to initiate an Arabidopsis ERAD process.

## Data Availability Statement

The original contributions presented in the study are included in the article/Supplementary Material; further inquiries can be directed to the corresponding authors.

## Author Contributions

JL conceived the research plans. JL and LinL supervised the project, suggested experiments, and wrote the article with supports from JM, JV, Y-MS, XP, and ZH. YX initiated, JZ continued, and DW completed the project with technical supports from YC, CZ, and MW. YD performed the exomannosidase digestion experiments with supervision from LiL and JV. Y-MS carried out the mass spectrometry assays. JZ, DW, YX, YD, JV, LinL, and JL analyzed the data and prepared figures. All authors contributed to the article and approved the submitted version.

## Funding

This study was supported partly by grants from the National Natural Science Foundation of China (NSFC31730019 to JL and NSFC31600996 to LinL), a grant from the National Science Foundation (IOS1121496 to JL), and a grant from the Chinese Academy of Sciences (2012CSP004 to JL).

## Conflict of Interest

The authors declare that the research was conducted in the absence of any commercial or financial relationships that could be construed as a potential conflict of interest.

## Publisher’s Note

All claims expressed in this article are solely those of the authors and do not necessarily represent those of their affiliated organizations, or those of the publisher, the editors and the reviewers. Any product that may be evaluated in this article, or claim that may be made by its manufacturer, is not guaranteed or endorsed by the publisher.

## Footnotes

^1^ www.treedyn.org
