# Supplementary material for: A Predominant Role of AtEDEM1 in Catalyzing a Rate-Limiting Demannosylation Step of an Arabidopsis Endoplasmic Reticulum-Associated Degradation Process
Source: Front Plant Sci. 2022 Jul 7;13:952246. doi: 10.3389/fpls.2022.952246 (PMC9302962; doi:10.3389/fpls.2022.952246)
Supplement: Supplementary file 1 [file Data_Sheet_1.PDF]

Supplemental Figures and Table

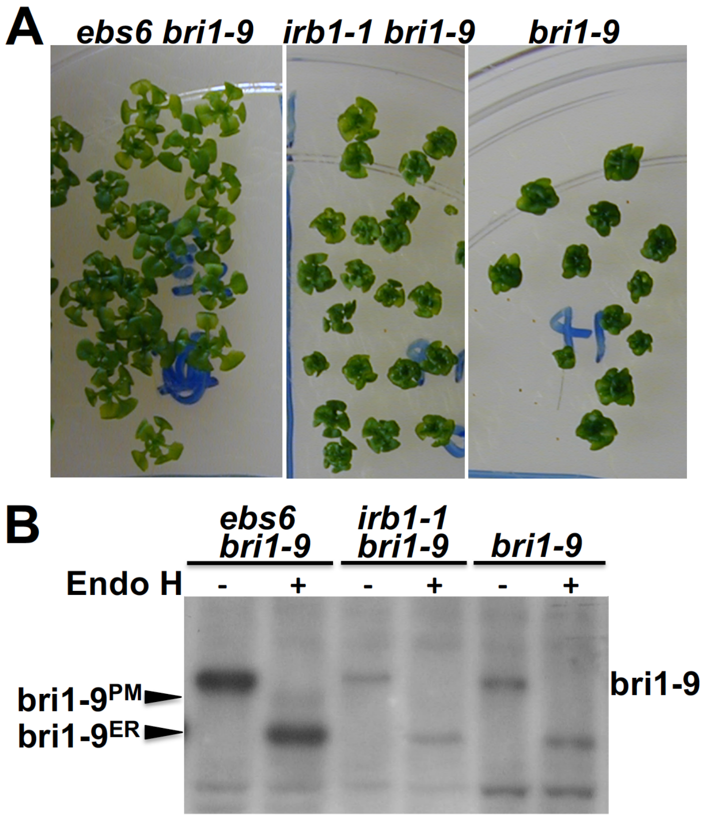

Supplemental Figure 1. The *irb1-1* mutation fails to suppress the *bri1-9* dwarfism and to inhibit bri1-9 degradation.

(A) Photographs of two-week-old petri dish-grown seedlings. **(B)** Immunoblot analysis of the bri1-9 protein abundance. Total proteins extracted from 2-week-old petri dish-grown seedlings were treated with or without Endo Hf, separated by SDS-PAGE, and analyzed by anti-BRI1 antibody.

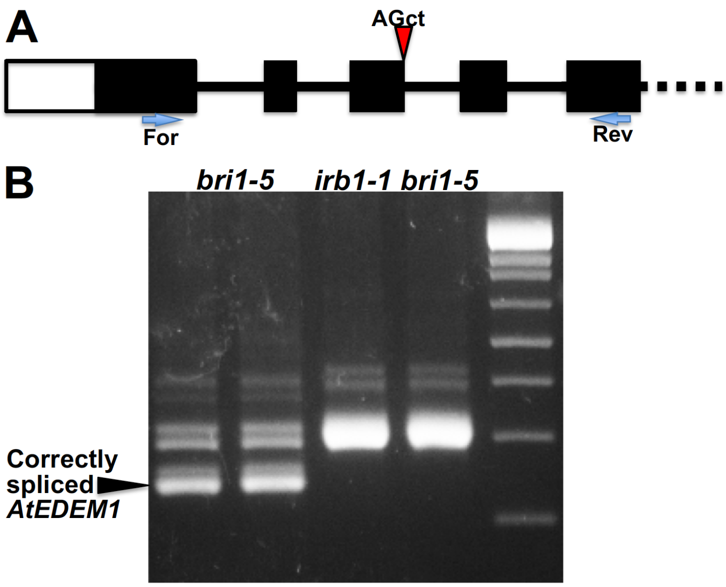

Supplemental Figure 2. The *irb1-1* mutation causes a splicing defect of *AtEDEM1* mRNA. **(A)** A diagram of the first 5 exons and introns of the *AtEDEM1* gene. The black line indicates intron while black bars represent exons. The red arrow shows the location of the *irb1-1* mutation that changes the "AG/gt" EXON/intron junction to "AG/ct" while the blue arrows indicate the two primers used for RT-PCR analysis of the *AtEDEM1* transcripts. **(B)** Gel electrophoresis of the RT-PCR analysis of the *AtEDEM1* transcripts using the first-strand cDNAs derived from total RNAs isolated from 2-week-old seedlings of *bri1-5* and *irb1-1 bri1-5* mutants. The arrow indicates the correctly-spliced *AtEDEM1* mRNA, which was absent in the *irb1-1 bri1-5* double mutant.

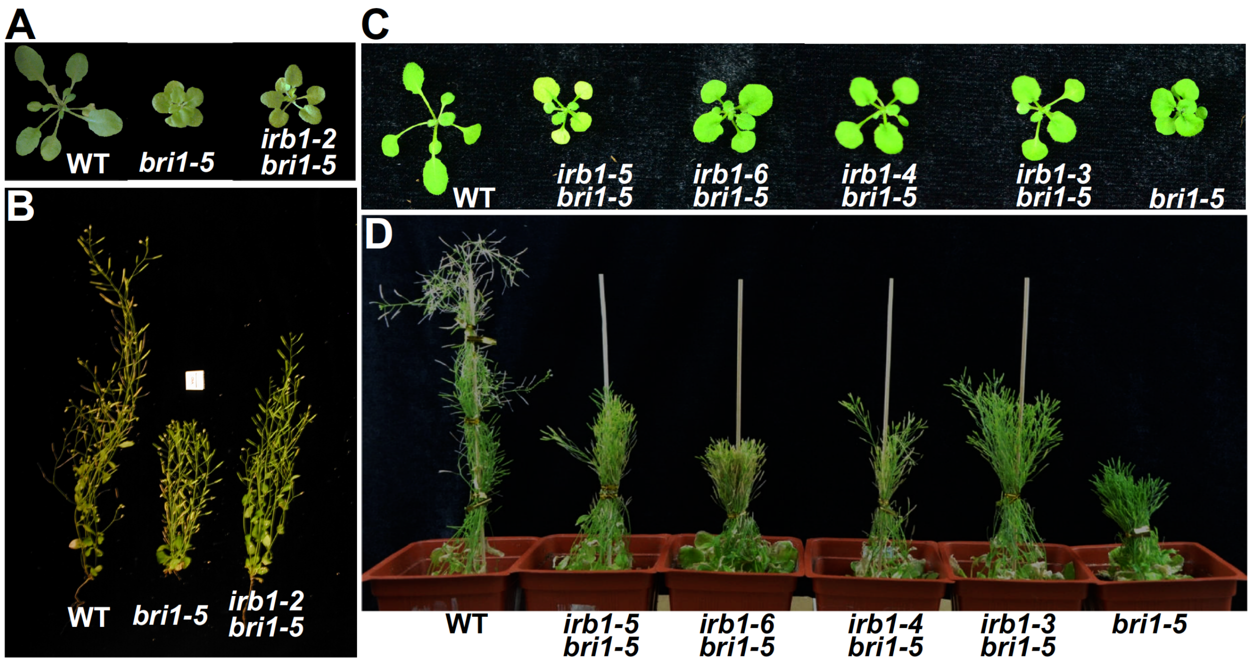

Supplemental Figure 3. Identification of 5 additional *irb1 bri1-5* mutants.

(A,C) Photographs of 2-week-old light-grown seedlings. **(B,D)** Photographs of mature soil-grown plants of the corresponding mutants shown in **A** and **C**, respectively.

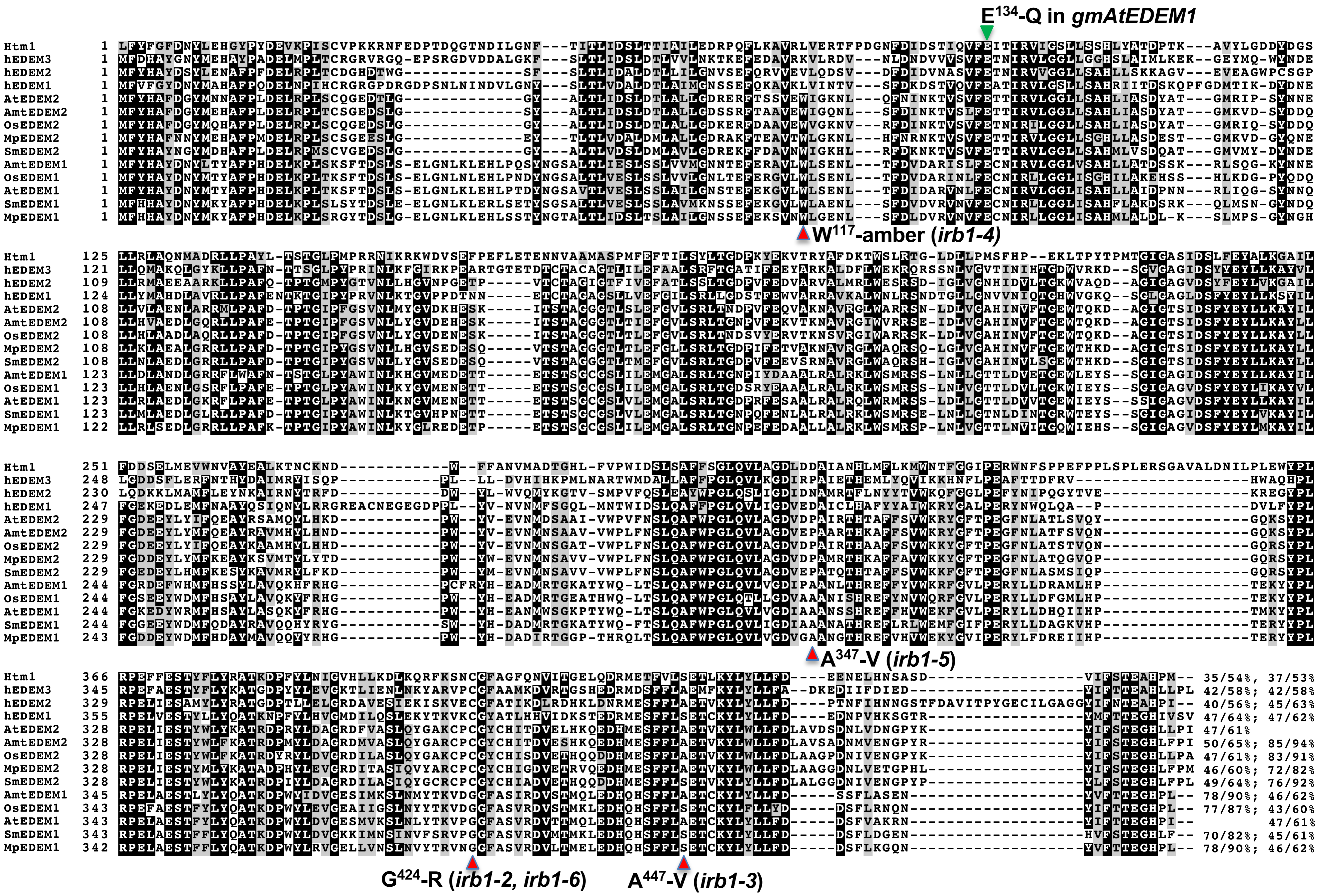

E134-Q in gmAtEDEM1
W117-amber (irb1-4)
A347-V (irb1-5)
G424-R (irb1-2, irb1-6)
A447-V (irb1-3)

Supplemental Figure 4. Sequence alignment of AtEDEM1/AtEDEM2 with their homologs. Amino acid sequences were downloaded from GenBank (see "**MATERIALS AND METHODS"** for their accession numbers) and aligned with the MUSCLE program at www.phylogeny.fr. This initial alignment was used to identify the conserved glycosylhydrolase core of 430 amino acids for each analyzed sequence, which were aligned again using the CLUSTALW program at https://www.genome.jp/tools-bin/clustalw, and the newly aligned sequences were visualized by the BoxShade program at https://embnet.vital-it.ch/software/BOX_form.html. The green arrow shows the absolutely-conserved glutamate residue that was mutated in a mutant genomic transgene of *gmAtEDEM1* used to study the requirement of its suspected α1,2-mannosidase activity, while the red arrows show the amino acids mutated in 5 other *irb1* mutants shown in **Supplemental Figure 3**. The percentages shown at the end of aligned sequences are percentage of sequence identity/similarity of the yeast Htm1, three human EDEMs, and selected plant EDEM homologs with AtEDEM1 (on the left) and AtEDEM2 (on the right).

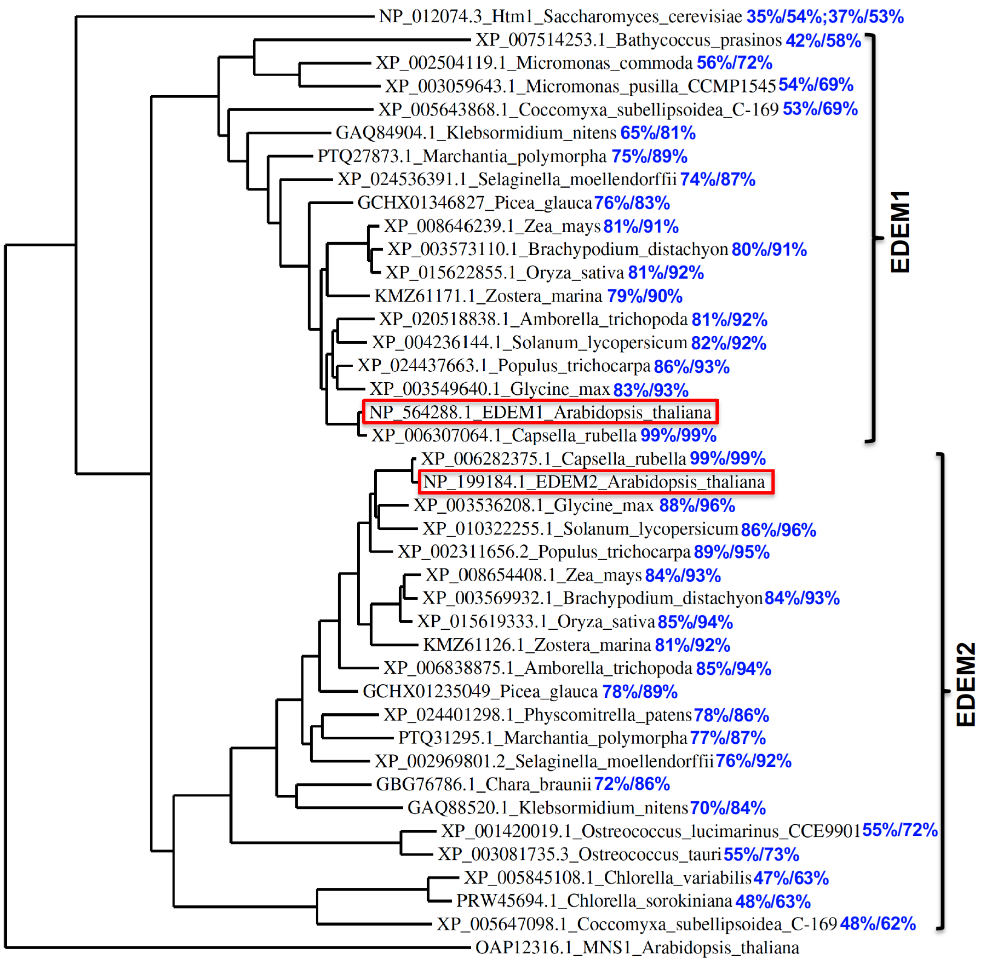

Supplemental Figure 5. Phylogeny of green lineage EDEMs.

Amino acid sequences (their accession numbers shown along with the species names) were downloaded from GenBank and were aligned with the MUSCLE program at www.phylogeny.fr. The aligned sequences were subsequently used to calculate the phylogeny tree with PHYML program at www.phylogeny.fr using the bootstrap procedure (number of bootstraps: 100). The resulting tree was subsequently visualized using the TreeDyn program (www.treedyn.org) at www.phylogeny.fr. The percentages shown (in blue color) after the species names are percentages of sequence identity/similarity between AtEDEM1 or AtEDEM2 with their plant homologs within the highly-conserved 430-amino-acid core. The amino acid sequences of the yeast Htm1 (its percentages of sequence identity/similarity with AtEDEM1/AtEDEM2 also shown) and the Arabidopsis MNS1 were used as outgroups to root the phylogeny tree.

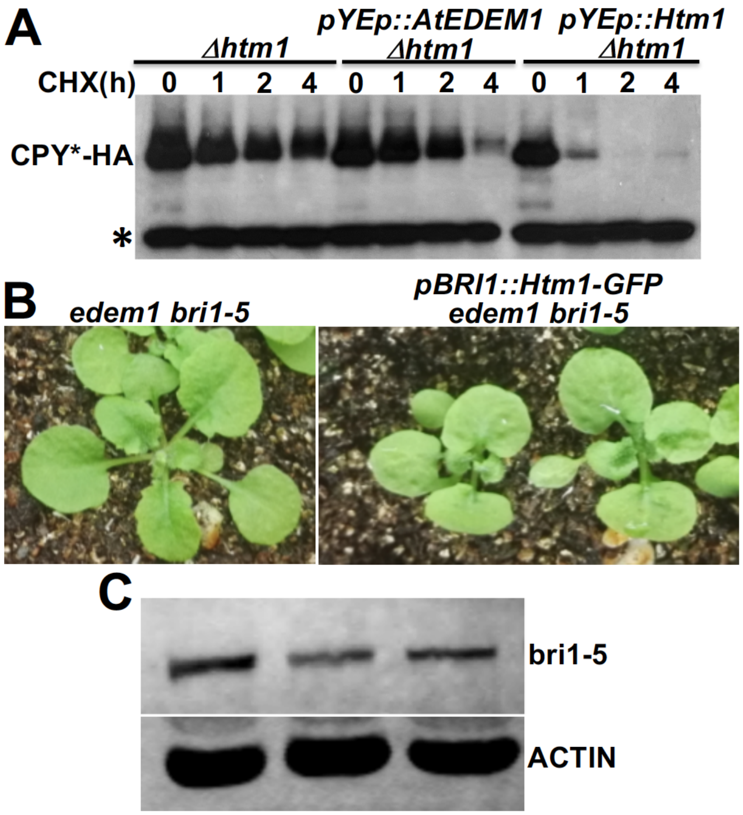

Supplemental Figure 6. AtEDEM1 is a functional homolog of the yeast Htm1.

(A) Immunoblot analysis of the HA-tagged CPY*, a widely-used yeast ERAD substrate. Total proteins extracted from equal amounts of the mid-log phase *Δhtm1* cells (transformed and non-transformed) treated with or without 180 μM CHX were separated by SDS-PAGE and analyzed by immunoblotting with anti-HA antibody. The star sign indicates a cross-reacting band used as a loading control. **(B)** Photographs of 4-week-old soli-grown plants of *irb1-1 bri1-5* and *pBRI1::Htm1-GFP irb1-1 bri1-5*. **(C)** Immunoblot analysis of the bri1-5 protein abundance with total proteins extracted from plants shown in (**B**) with antibodies to BRI1 and ACTIN (as a loading control).

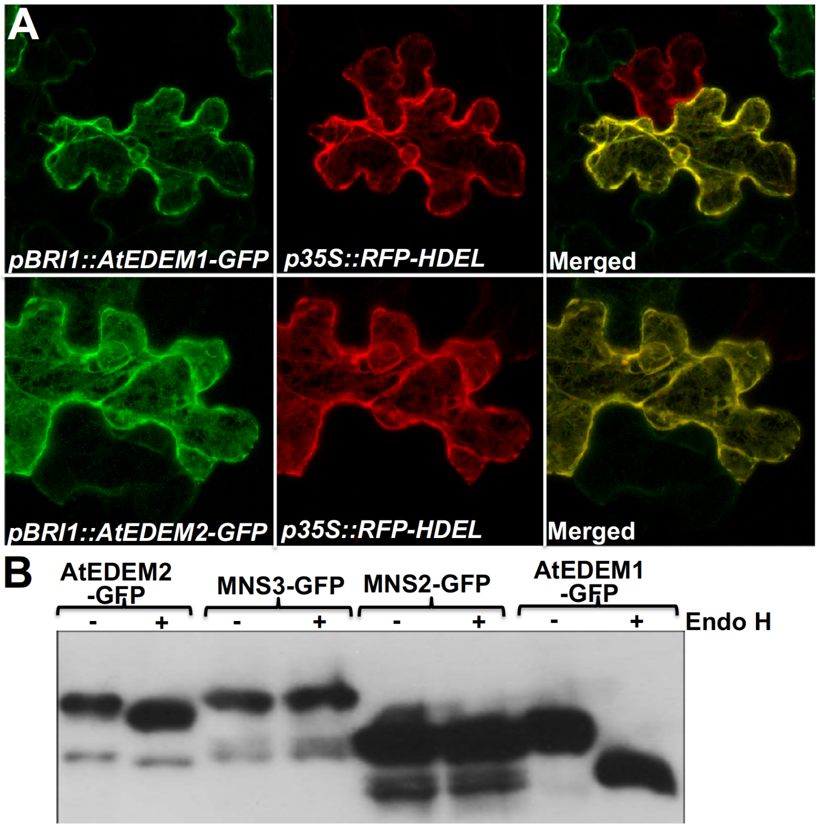

Supplemental Figure 7. Both AtEDEM1 and AtEDEM2 are ER-localized proteins.

(A) Confocal microscopic examination of the GFP-tagged AtEDEM1 (top row) and ATEDEM2 (bottom row) that were transiently expressed in tobacco leaves along with a widely used ER marker, RFP-HDEL. Shown here are the GFP images (on the left), the RFP images (in the middle), and the superimposed images of GFP and RFP signals (on the right). **(B)** Endo H analysis of the tobacco leaf-expressed GFP fusion proteins of ATEDEM1/2 along with GFP-tagged MNS3 (At1g30000, an Arabidopsis homolog of the mammalian ER mannosidase 1) and MNS2 (At1g51590; one of the two Arabidopsis homologs of the mammalian Golgi-localized α1,2-mannosidases).

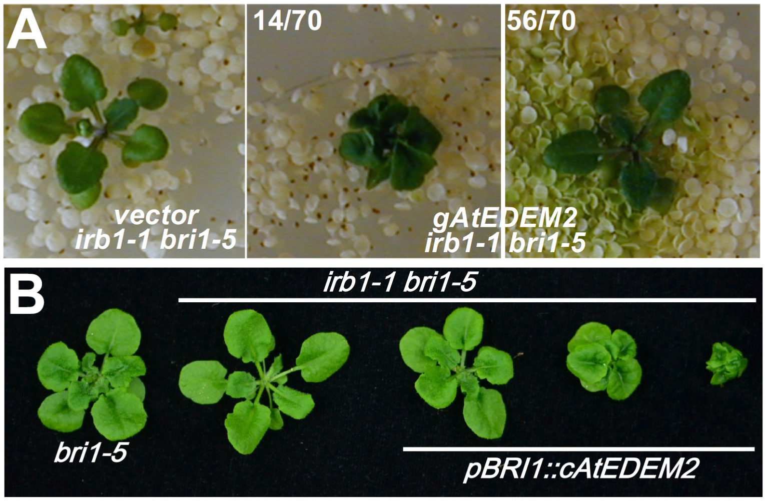

C

	rescue	strong	severe
pBRI1::EDEM1/irb1-1 bri1-5	**14**	**16**	**50**
pBRI1::EDEM2/irb1-1 bri1-5	**11**	**36**	**35**

Supplemental Figure 8. AtEDEM2 likely plays a minor role in bri1-5 degradation.

(A) Phenotypes of T1 transgenic *irb1-1 bri1-5* lines transformed with a genomic construct of *AtEDEM2* and a vector control. Shown are numbers of transgenic lines that were morphologically similar to *bri1-5* (in the middle) or to the parental *irb1-1 bri1-5* mutant (on the right) out of a total 70 transgenic lines. **(B)** Phenotypic grouping of transgenic *pBRI1::cAtEDEM1/2 irb1-1 bri1-5* mutants that were grown in soil for 4 weeks. **(C)** Number of transgenic *pBRI1::AtEDEM1/AtEDEM2 irb1-1 bri1-5* mutants exhibiting similar growth morphology to the Arabidopsis lines shown in (**B**).

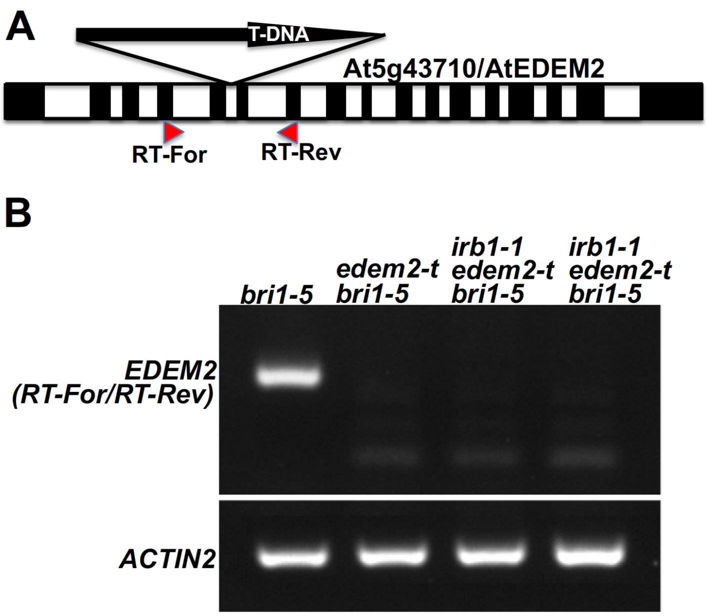

Supplemental Figure 9. The *edem2-t* mutant is likely a null mutant.

(A) A diagram of the T-DNA insertion in the *AtEDEM2* gene. The black arrow indicate the position and orientation of the inserted T-DNA. Black bars represent exons, white boxes denote introns, red arrows stand for primers used for analyzing the *AtEDEM2* transcript abundance (RT-For/RT-Rev). **(B)** Two gel images of RT-PCR products of *AtEDEM2* (upper image) and *ACTIN2* (lower image). Total RNAs were extracted from 2-week-old petri dish-grown Arabidopsis seedlings of *bri1-5*, *edem2-t bri1-5*, and *irb1 edem2-t bri1-5* mutants and converted into first-strand cDNAs, which were used as the template to amplify the cDNA fragments of *AtEDEM2* or *ACTIN2* using the primer sets listed in Supplemental Table 1. The amplified cDNA fragments were separated by agarose gel electrophoresis, stained with ethidium bromide, and photographed with a Gel Documentation system.

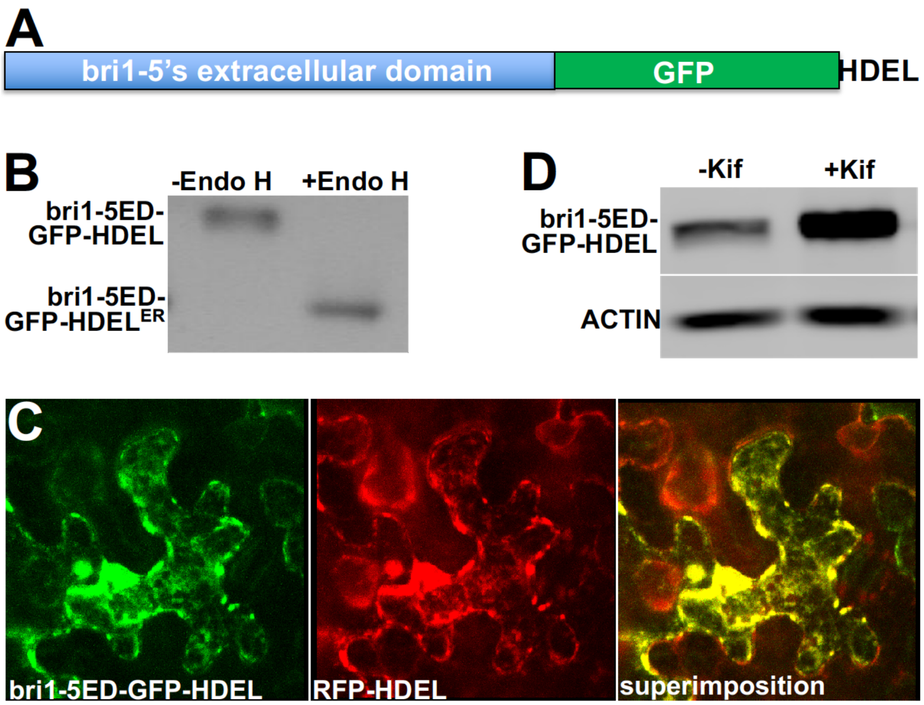

Supplemental Figure 10. The bri1-5ED-GFP-HDEL is an ERAD substrate.

(A) A diagram showing the domain organization of the bri1-5ED-GFP-HDEL fusion protein. **(B)** Endo H analysis of the transgenically expressed bri1-5ED-GFP-HDEL. **(C)** Confocal microscopic examination of the bri1-5ED-GFP-HDEL that was transiently expressed in the tobacco leaf epidermal cells. Shown here are the green image of bri1-5-GFP-HDEL (left), the red image of the widely-used ER marker RFP-HDEL (in the middle), and the superimposed image of red and green channels (right). **(D)** Immunoblot analysis of the bri1-5ED-GFP-HDEL from 2-week-old seedlings treated with or without 5 μM kifunensine (Kif) with anti-BRI1 (upper strip) and anti-ACTIN (lower strip, serving as a loading control) antibodies.

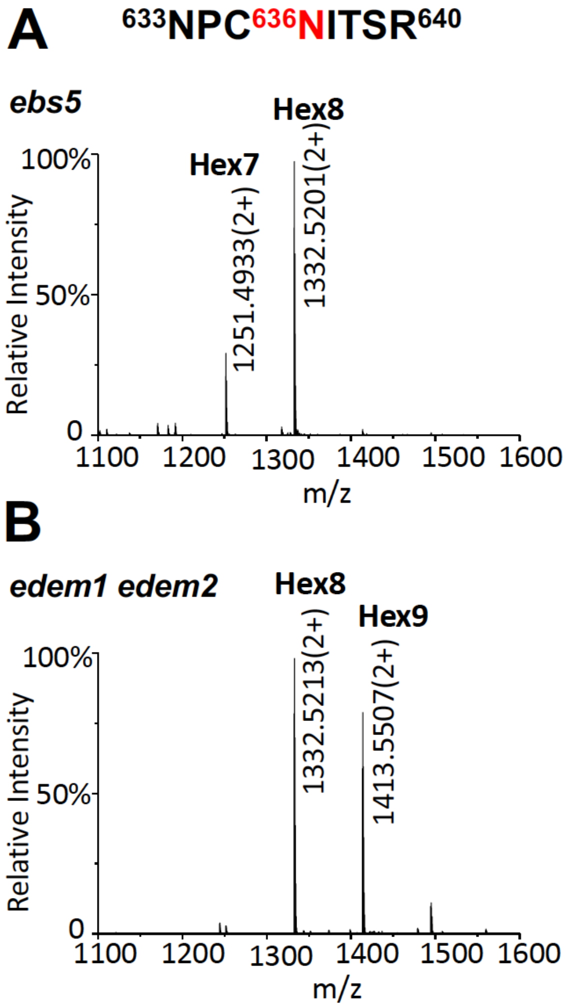

Supplemental Figure 11. Mass spectrometric analysis of another glycosylated peptide of bri1-5ED-GFP-HDEL.

(A) Mass spectrum of the glycosylated peptide at residues 633-640 derived from a tryptic digestion of the immunoprecipitated bri1-5ED-GFP-HDEL of the *ebs5 bri1-5* mutant. The glycopeptides at the doubly charged ions of m/z 1251.4933 and 1332.5201 were identified to contain two high mannose-type N-glycans of Hex7 and Hex8 by MS/MS measurements. **(B)** Mass spectrum of the glycosylated peptide at residues 633-640 derived from a tryptic digestion of the immunoprecipitated bri1-5ED-GFP-HDEL of the *irb1-1 edem2-t bri1-5* mutant. The glycopeptides at the doubly charged ions of m/z 1332.5213 and 1413.5507 were identified to contain two high mannose-type N-glycans of Hex8 and Hex9 by MS/MS measurements.

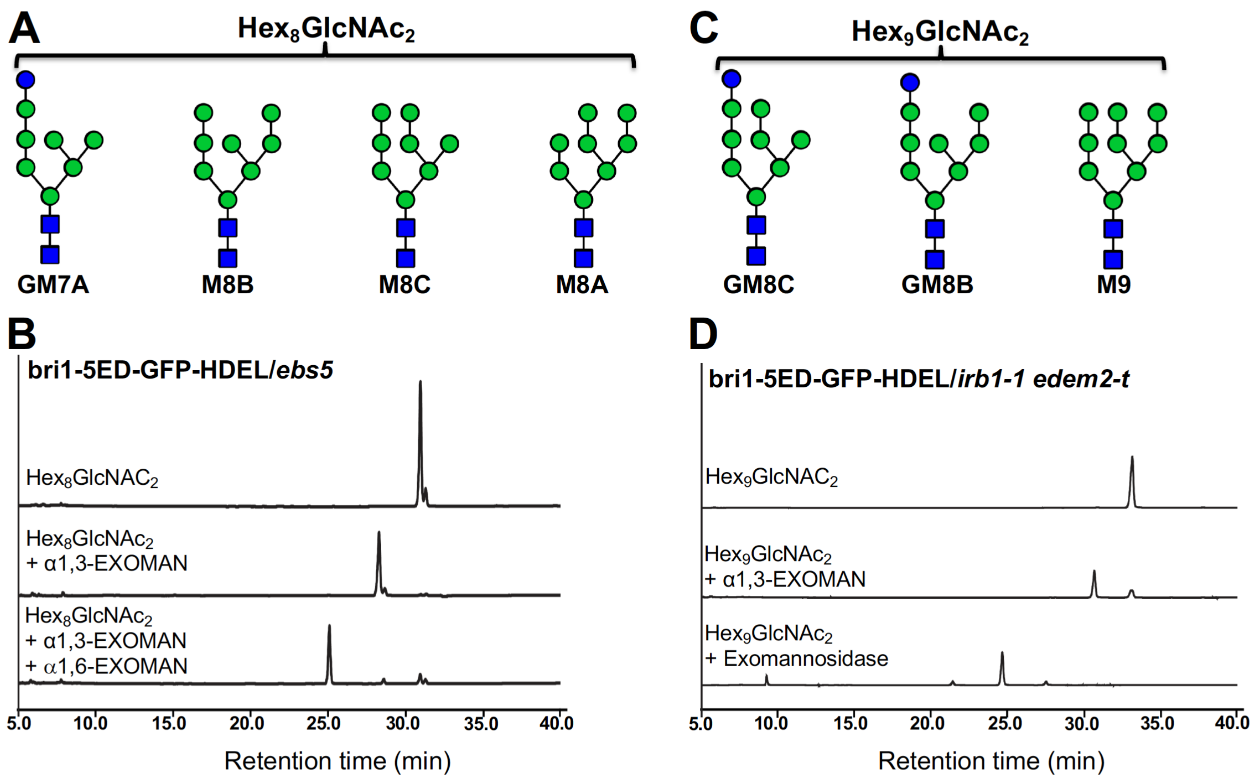

Supplemental Figure 12. Structural analysis of N-glycans using exomannosidases.
(A,C) Possible N-glycan structures of $Hex_8GlcNAc_2$ and $Hex_9GlcNAc_2$. The structure of $GlcMan_7GlcNAc_2$ (**GM7A**) is the only glycan sensitive to both α1,3- and α1,6-exomannosidases, while $GlcMan_8GlcNAc_2$ (**GM8B**) is the only glycan sensitive to α1,3-mannosidase. Green circles denote mannose residues, blue circles indicate glucose residues, while square boxes represent N-acetyl glucosamine residues. The vertical line linking two mannose residues represents the α1,2 linkage, the 2-o'clock lines denote the α1,6 linkage, and the 10-o'clock lines indicate the α1,3 linkage. **(B,D)** LC chromatograms of the exomannosidase-digested $Hex_8GlcNAc_2$ (**B**) and $Hex_9GlcNAc_2$ (**D**) glycans released from immunoprecipitated bri1-5ED-GFP-HDEL of *ebs5 bri1-5* (**B**) and *irb1-1 edem2-t bri1-5* (**D**) mutants, respectively. Dual sensitivity of the $Hex_8GlcNAc_2$ peak to α1,3 and α1,6-exomannosidases indicates its structural identity to the **GM7A** form of N-glycan shown in (**A**). The α1,3-exomannosidase sensitivity of the $Hex_9GlcNAc_2$ peak reveals its structural identity to the **GM8B** glycoform shown in (**C**). The released $Hex_9GlcNAc_2$ glycan from the bri1-5ED-GFP-HDEL of the *irb1-1 edem2-t bri1-5* triple mutant is partially sensitive to α1,2/3/6-exomannosidase that should sequentially remove all mannose residues (α1,2-/α1,3-/α1,6-mannose residues) except the GlcNAc-linked one. This partial mannosidase sensitivity indicates the presence of a glucose residue at the A branch of **GM8B,** which renders the two α1,2-mannose residues resistant to cleavage by the α1,2/3/6-exomannosidase.

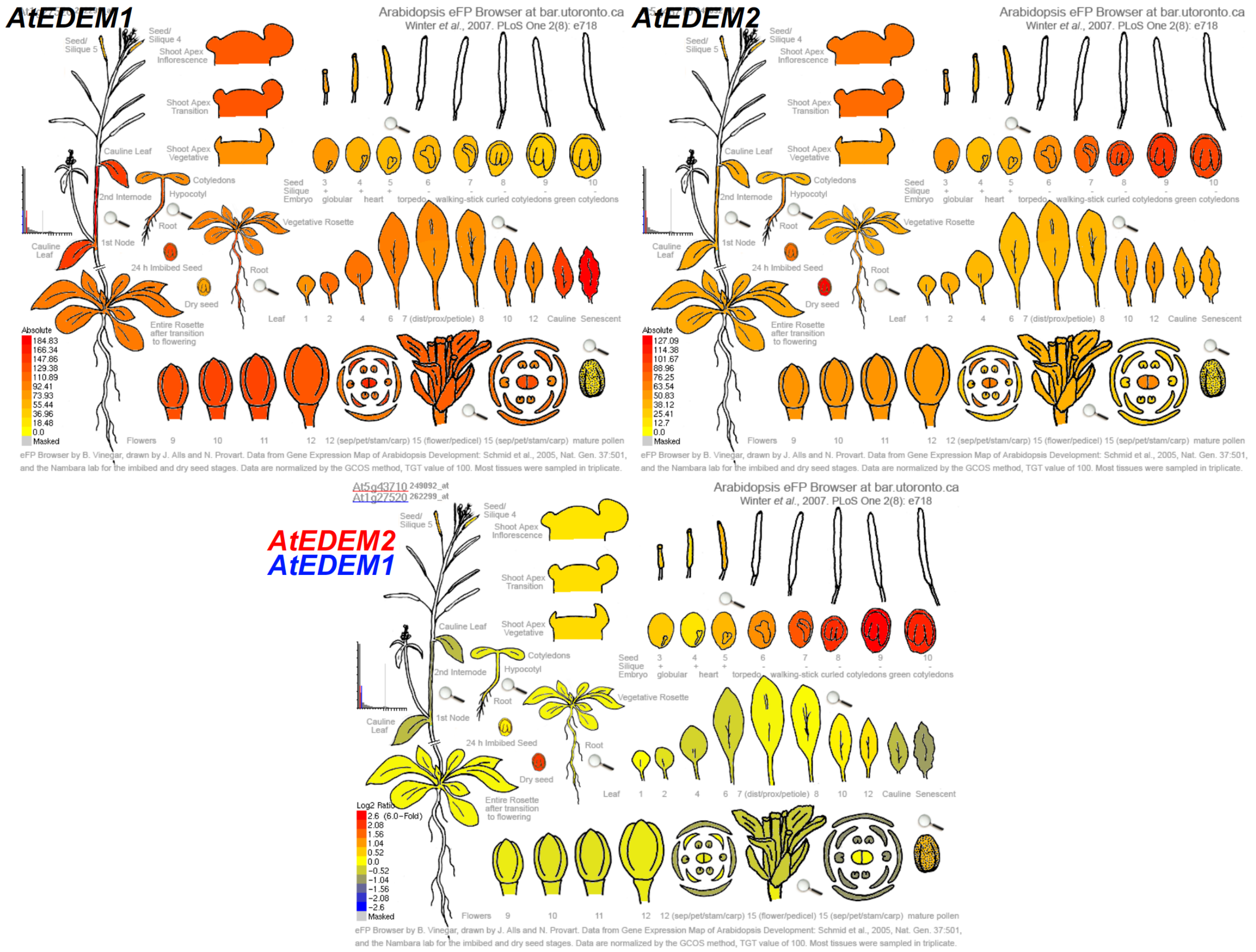

Supplemental Figure 13. Expression patterns of *AtEDEM1* and *ATEDEM2.*

The top two diagrams reveal the overlapping expression patterns of *AtEDEM1* (*At1g27520* on the left) and *AtEDEM2* (*At5g47310* on the right) in different tissues of growing Arabidopsis plants. The bottom figure indicate the expression difference between the two homologous genes, revealing higher expression of *AtEDEM2* in developing/mature seeds and slightly higher expression of *AtEDEM1* in floral organs and senescing leaves. All three figures were obtained from the Arabidopsis eFP browser web site http://bar.utoronto.ca/efp_arabidopsis with reprint permission from a previously published study [1].

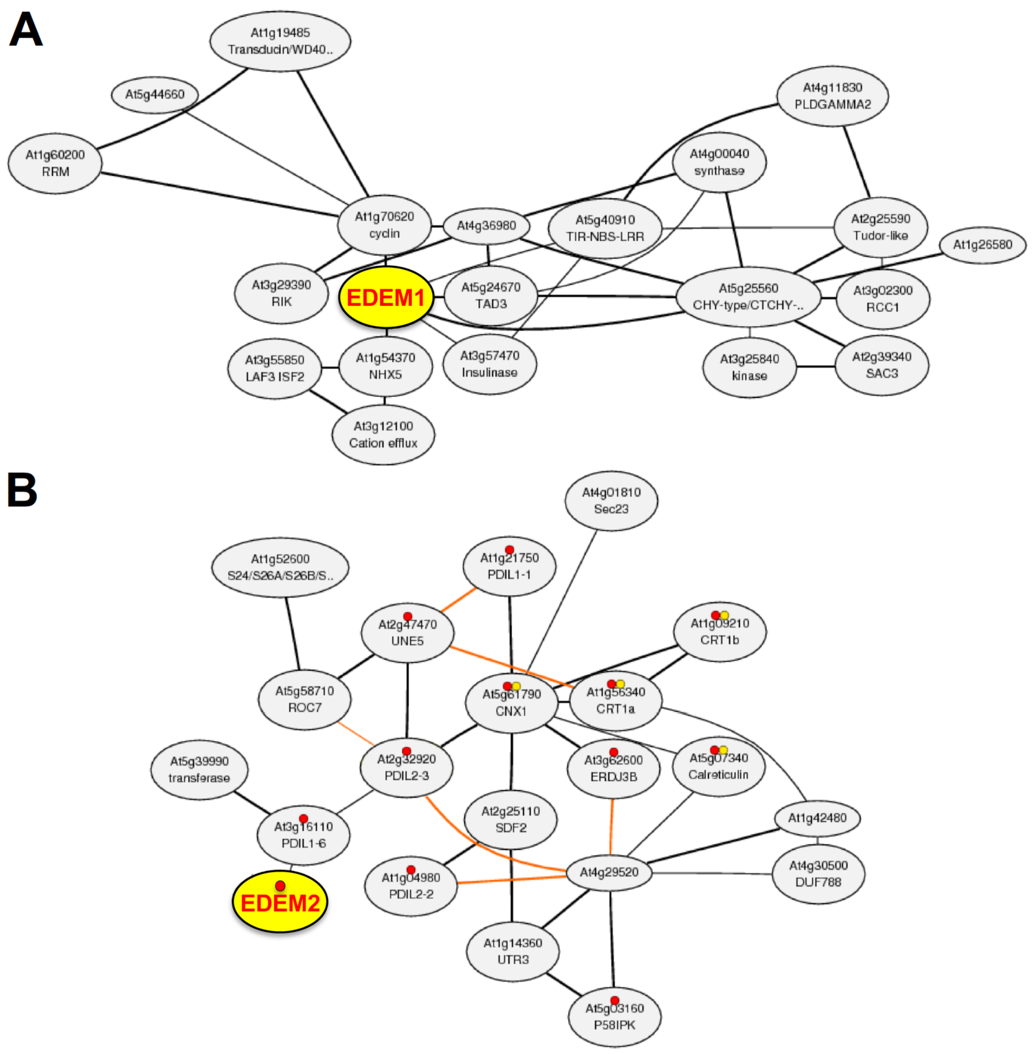

Supplemental Figure 14. *AtEDEM1* and *AtEDEM2* exhibit different gene coexpression profiles. (A,B) Shown in the figures are genes that were shown by multiple microarray/RNA-seq-based transcriptome studies to be coexpressed with *AtEDEM1* (**A**) or *AtEDEM2* (**B**). The two figures were generated by performing searches at ATTEDII (atted.jp) [2] using the gene IDs (*At1G27520* or *At5g43710*) as the query. The two images were obtained from the locus pages of the search results. Red and yellow dots denote proteins known or predicted to be involved in protein processing in the ER-mediated protein processing and phagosome, respectively. Black thick and thin lines link strongly and weakly coexpressed genes, respectively, while orange lines denote coexpressed gene orthologs in other living organisms.

Supplemental Reference

1. Winter, D.; Vinegar, B.; Nahal, H.; Ammar, R.; Wilson, G. V.; Provart, N. J., An "electronic fluorescent pictograph" browser for exploring and analyzing large-scale biological data sets. *PLoS One* **2007,** 2, (1), e718.
2. Obayashi, T.; Hayashi, S.; Saeki, M.; Ohta, H.; Kinoshita, K., ATTED-II provides coexpressed gene networks for Arabidopsis. *Nucleic Acids Res* **2009,** 37, (Database issue), D987-91.

Supplemental Table S1. Oligonucleotides used in this study

Primer Name	Sequence		Note
	Forward primer	Reverse primer	
**RT-PCR primers**			
AtEDEM1	ATCGCAGCATATCGAGGTCAA	CGGGCATCAATATCGAAAGTAAGA	analyzing the defect of *irb1-1* mutation
AtEDEM2	TTACTATCTGCTCATCTGATTGCA	AAACGACTAAGCACGCCAAACTC	analyzing the effect of the *edem2-t* mutation
ACTIN2	CGTACAACCGGTATTGTGCT	GATGTCTCTTACAATTTCCCGCT	the internal reference for RT-PCR analysis
**Making constructs**			
BRI1ED	GGA**ACTAGT**GCTTCCACTTCCTCTCTAA	CGC**GGATCC**TCCTTCCATGAGATCT	cloned into the *pBRI1::BRI1-GFP* plasmid
GFPHdel	AG**GGATCC**CATGAGTAAAGGAGAAGAAC	TC**GGTACC**CTATAATTCATCATGTTTGTATAGTTCATCCATGC	cloned into the *pBRI1::BRI1ED-GFP* plasmid
c1g30000GFP	GC**TCTAGA**GAGAAATGTCGAAATCTCTACC	GC**GGATCC**GTGTTTCTTCTTATTGGTAATG	cloned into the *pBRI1::BRI1-GFP* plasmid
c1g51590GFP	GG**ACTAGT**GAGAAATGGCGAGAAGTAGATCG	GC**GGATCC**ACGTTAATCTGATGACCAAAC	cloned into the *pBRI1::BRI1-GFP* plasmid
cAtEDEM1GFP	GG**ACTAGT**CGGAGACTGGCGGAGATGATGA	GC**GGATCC**AAGGGATTCCATAAGCCGCAG	cloned into the *pBRI1::BRI1-GFP* plasmid
cAtEDEM2GFP	GG**TCTAGAG**AGAAATGGACTCAAATTTCAAGTG	GC**GGATCC**GAAGAGTAGATCTTTGATC	cloned into the *pBRI1::BRI1-GFP* plasmid
Htm1GFP	GG**ACTAGT**GAGAAATGGTTTGCTGCTTATGG	CT**TCTAGA**ACAATAAATAAGTTGATGATGGGCG	cloned into the *pBRI1::BRI1-GFP* plasmid
pBRI1AtEDEM1	CTCACAA**GGATCC**ATGTCTTGTCCTATCCATCC	GAATTG**GGTACC**TAAGGGATTCCATAAGCCGC	cloned into the *pC1300BRI1* plasmid
pBRI1AtEDEM2	CTCACAA**GGATCC**ATGGACTCAAATTTCAAGTGG	GAATTG**GGTACC**AGAAGAGTATGATCTTTGATC	cloned into the *pC1300BRI1* plasmid
gAtEDEM1	CCG**CTCGAG**AAGCAATCCTTGATAAGT	CGC**GAATTC**GGATGCGTCTACCCTAT	5,279bp PCR fragment cloned into *pPZP222*
gAtEDEM2	GC**CCCGGG**AGTTCAAGCGATGAAG	GC**GGTACC**AATCTATCGAGCAGTGAG	6,323bp PCR fragment cloned into *pPZP222*
pYEP352EDEM1	ATGTCTTGTCCTATCCATCCTAGG	GG**ACTAGT**CTATAAGGGATTCCATAAGC	cloned into *pYEp352-ScALG9*
pYEP352Htm1	ATGGTTTGCTGCTTATGGG	GG**ACTAGT**CTATACAATAAATAAGTTGATG	cloned into *pYEp352-ScALG9*
AtEDEM1Mut	CGGGTCAACCTTTTT**C**AGGTAGGAGTCTAGTTCTG	CAGAACTAGACTCCTACCT**G**AAAAAGGTTGACCCG	
bri1-5Mut	CTTTCGATGGCGTTACTT**A**CAGAGACGACAAAGTTAC	GTAACTTTGTCGTCTCTG**T**AAGTAACGCCATCGAAAG	
**Genotyping/Mapping**			
irb1-1	TGCATTTCCGGTTAGTGATCTAC	TGATGCTATATGAGCTAGTACAAAGATA	EcoRV cuts the mutant fragment
Alg3 (046061)	TTTGCTTGCGTATCCCTCG	GTAGACTTCCCCTCCAGTTA	Lba1-Rev 87bp; For+Rev, 313 bp
5669k(SSLP)	GACGAGTTACTTACAATAATTGATC	GATTTCCATTGTGTCAACTTGCTACC	Col, 258bp; Ws-2, 220bp
5669k(HindIII)	GTACACGCTTGAAGGGCCATTTAC	TGGTCCATGGTACATCCAAGAAGCT	dCAPS marker, HindIII cuts the Col fragment
edem2-t	GTGATTATGCAACGGTACGT	TGTTTGTCAACACCGTACATC	*SALK_095857* (Lba1+Rev)
9343k	CACTGCTACATGTATATATGTTCTC	AATGGTGGGTTGCCACCTAATCTGA	Col, 231bp; Ws-2, 211bp
9877k	CCATTTCCATGAAAGAGCTCTCGATGC	TGGCTCCTCGGCTCCCATACATGCACTT	NsiI cuts the Ws-2 PCR fragment

11160k	GTACGATGGTTCAATGGACTAACCAAGT	CTGTCCCGGCGAGTGGCCGTTTTAGT	Col-0, 304bp; Ws-2, 204bp
11160k(HindIII)	CCTCGTAGGCAACCAGTCGTAGTAA	GGGAGAAAAAGCTGACATGGAGAAGCT	HindIII cuts the Ws-2 PCR fragment
F15O4	ATCAAAGGTTGCCACAAATG	TTCGGTTTGATTTGGGTTTC	Col-0, 236bp; Ws-2, 250bp
ciw12	AGGTTTTATTGCTTTTCACA	CTTTCAAAAGCACATCACA	SSLP, Ws-2<Col-0
LBa1	TGGTTCACGTAGTGGGCCATCG		For genotyping *alg3-t2* and *edem2-t*
AtEDEM1EQ	AAGTCCTCCAAGAACTCTTATATTGAGCT	GCTTGAACACCTGCCAACAGATTA	SacI cuts the WT PCR fragment
bri1-5	CCGTGTACTTTCGATGGCGTTACCT	CCAAGCTGGTTAAAGAAGCAGAGCA	PstI cuts the WT PCR fragment
bri1-9	ATGTATCCAGACAACATGTTTTAA	GGCTTGATCTCAACACCA	DraI (dCAPS) cuts the mutant fragment
